# Wafer‐Scale Bandgap‐Tunable MoS_2_/PbS Phototransistors Enabled by Solution Processing

**DOI:** 10.1002/advs.202518844

**Published:** 2026-01-12

**Authors:** Ziheng Tang, Chengqian Cui, Xiaoli Jing, Rui You, Mingjun Zhang, Jing Xu

**Affiliations:** ^1^ Department of Mechanical Engineering Tsinghua University Beijing China; ^2^ School of Biomedical Engineering Tsinghua University Beijing China; ^3^ School of Instrument Science and Opto‐electronics Engineering Beijing Information Science and Technology University Beijing China

**Keywords:** bandgap engineering, heterostructures, low‐dimensional electronics, phototransistors, solution‐processing

## Abstract

Molybdenum disulfide (MoS_2_)/lead sulfide (PbS) heterostructures exhibit exceptional potential because of their strong light‐matter interactions and high carrier mobility. Critically, bandgap engineering can further optimize the light‐absorption range for next‐generation phototransistors. However, the bandgap engineering capability for MoS_2_/PbS heterojunctions formed by conventional transfer‐after‐chemical vapor deposition (CVD) fabrication is typically inherently restricted due to solely vertical interlayer coupling. Here, to realize wafer‐scale bandgap‐tunable MoS_2_/PbS phototransistors, we investigate the band structure of vertical and lateral MoS_2_/PbS heterojunctions via ab initio calculations and find that lateral heterojunctions in heterostructures dominate the bandgap tunability via tuning of the Type‐II band alignment. To achieve wafer‐scale uniformity, we investigated how plasma treatment modulates the thin‐film surface energy, and the results substantially improved fabrication scaling of MoS_2_/PbS heterojunctions from traditional micro‐scale level to an incredible 4‐inch wafer‐scale with near‐ideal yields (97%) and enabled bandgap tunability (from 1.24 to 0.61 eV). The resulting phototransistors exhibit a maximum responsivity of 88 A/W, specific detectivity of 4.77  ×  10^12^ Jones, and a typical on/off ratio of 3.16  ×  10^7^. This work establishes a pathway for developing wafer‐scale bandgap‐tunable optoelectronics.

## Graphical abstract

This work advances bandgap‐tunable MoS_2_/PbS phototransistors by introducing lateral heterojunctions, which enable superior tunable bandgap (1.24–0.61 eV) via Type‐II alignment. Plasma‐enhanced solution processing ensures uniform 4‐inch wafer‐scale fabrication with 97% yield. The optimized devices achieve high responsivity (88 A/W), detectivity (4.77 × 10^1^ ^2^ Jones), and on/off ratio (3.16 × 10^7^), providing a pathway for scalable, tunable optoelectronics.

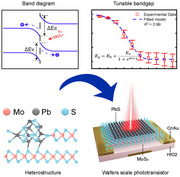

## 1 Introduction

Phototransistors play crucial roles in converting light signals into electrical signals and have been used in various applications, including optical communication, imaging, remote sensing, digital photography, and solar cell applications [1, 2, 3, 4, 5, 6, 7, 8]. Novel materials and fabrication techniques have been developed to fabricate wafer‐scale phototransistors with unique properties [9, 10, 11, 12, 13, 14]. In particular, over the past decade, 2D transition metal dichalcogenide (TMDC) materials and their heterostructures or superlattices have gained widespread attention and application in phototransistors because of their strong light‐matter interactions, high carrier mobility, and scalability [15, 16, 17]. Among these, MoS_2_ is a representative TMDC that exhibits a direct bandgap, a high carrier mobility (200 cm^2 ^V^−1 ^s^−1^), a high on/off ratio (1  ×  10^8^), and stands out as a promising candidate for future generation optoelectronics [18]. However, the bandgap of MoS_2_ (1.8 eV for monolayers) imposes substantial constraints on broader optoelectronic applications, as this bandgap results in the detectable spectral range being confined to the visible region (400–680 nm range [18]).

To broaden the detectable spectral range, heterostructures formed by assembling MoS_2_ with various materials have been explored [19, 20, 21, 22, 23, 24, 25]. Among these materials, PbS serves as a high‐performance light absorber across ultraviolet‐visible‐near‐IR (UV–Vis–NIR) spectra for optoelectronic applications [20]. However, most recent reports rely on CVD, where PbS is deposited atop CVD‐deposited highly compacted MoS_2_. This results in exclusively vertical architectures dominated by weak van der Waals interactions. Kufer et al. [19] reported the fabrication of MoS_2_/PbS heterojunctions through the decoration of PbS quantum dots (QDs) on MoS_2_ nanoflakes. The heterostructure extends the optical detection bandwidth, conferring superior infrared radiation photodetection capabilities. Similarly, Wang et al. [20] demonstrated epitaxial growth of PbS microplates on transferred MoS_2_ substrates via thermal evaporation of PbS powder, which enabled bandgap reduction and enhanced infrared photodetection performance. In these reported heterostructures, CVD‐synthesized MoS_2_ films are highly compacted and only exhibit (001) crystal facets [26]. Hence, the bandgap engineering capability of such transfer‐after‐CVD fabrication approaches is limited, as only the specific crystal facets of the highly compacted continuous films synthesized by CVD are exposed, thereby inducing heterojunctions with a fixed bandgap by the narrow‐gap material (PbS). Reliance on vertical architectures has fundamental limitations, including (1) restricted carrier transfer due to weak interfacial electric field (IEF) forces, (2) challenges in aligning the Fermi levels arising from weak van der Waals (vdW) gap, and (3) formation of Schottky barriers between heterostructures. Under such limitations, the heterojunction exhibits a Type‐I straddling band structure [20]. The band edges of the narrow‐gap semiconductor (PbS) lie entirely within the wider bandgap of the other material (MoS_2_). The tunability of the bandgap is fundamentally constrained by its dependence on the components, as the bandgaps of the constituents are inherently fixed. Therefore, bandgap engineering of conventional heterostructures is fundamentally constrained by vertical architectures, and the underlying mechanisms governing the bandgap tunability remain elusive.

To overcome these vertical limitations, recent research has pivoted toward lateral heterostructures. By stitching distinct 2D materials within the same atomic plane, lateral junctions can form Type‐II staggered band alignments that spatially separate electrons and holes [27], significantly enhancing carrier collection efficiency. Techniques such as lateral epitaxial growth and photolithography‐assisted patterning have successfully demonstrated lateral junctions with high detectivity and ultra‐fast response speeds [28]. However, these approaches—primarily relying on CVD or mechanical exfoliation—face intrinsic limitations in scalability and uniformity, making high‐yield wafer‐scale integration difficult. What's more, these often suffer from scalability issues as epitaxial growth requires precise lattice matching and often yields limited domain sizes [29, 30]. Furthermore, these methods typically result in fixed band alignments dictated by the specific material combination, lacking the flexibility for continuous bandgap engineering. Crucially, achieving continuous bandgap tunability in these lateral systems remains elusive, as the bandgap is typically fixed by the specific material combination used.

Besides CVD, an alternative heterostructure fabrication method is solution processing after electrochemical intercalation of 2D materials [31, 32, 33, 34, 35], in which materials inks are spin‐coated onto wafers to form thin semiconductor films. The solution‐processing technique introduces a new dimension of structural complexity and diversity, which potentially leads to novel electronic and optical properties in these mixed heterostructures [36, 37, 38]. Lateral structures emerge when QDs connect across the horizontal plane of MoS_2_ nanosheets, whereas hybrid structures form when QDs are positioned at the edges and steps of MoS_2_ layers. Therefore, the solution processing fabrication technique facilitates the creation of heterostructures with distinctive characteristics, setting them apart from their traditional counterparts. However, during heterostructure formation, the interfacial energy mismatch between thin films impedes the fabrication of wafer‐scale homogeneous via spin‐coating and induces crack propagation in the subsequent coated thin films, hindering wafer‐scale heterostructure fabrication. Hence, the solution methods for fabricating wafer‐scale phototransistors still need to be improved.

In this study, to realize bandgap‐tunable MoS_2_/PbS heterostructures for wafer‐scale phototransistors, we investigated MoS_2_/PbS heterostructures with distinct architectures through ab initio calculations and band structure analysis. We found that lateral heterojunctions dominate the bandgap tunability via tuning of the band alignment. PbS QDs infiltrate the intersheet gaps of solution‐processed MoS_2_ nanosheets, forming lateral heterojunctions via this incorporation process. This architecture enables a Type‐II staggered alignment, allowing us to tune the bandgap dynamically by adjusting the layer cycles, a capability absent in fixed‐bandgap vertical counterparts. While solution processing is known for scalability, it historically suffers from high defect densities and crack propagation due to interfacial energy mismatches between distinct 2D materials. Simple spin‐coating often leads to dewetting or aggregation, preventing wafer‐scale homogeneity. Our work distinguishes itself by introducing a plasma‐enhanced surface energy modulation technique. This optimized solution processing enabled fabrication of MoS_2_/PbS heterojunctions on 4‐inch wafers with near‐ideal yields (97%) and tuning of the bandgap from 1.24 to 0.61 eV. By employing the plasma‐enhanced solution‐processing method with metal–oxide–semiconductor field effect transistors (MOSFETs), we fabricated phototransistors exhibiting exceptional electrical properties and demonstrated their superior optoelectronic performance, with a highest responsivity of 88 A/W, specific detectivity of 4.77  ×  10^12^ Jones and a typical on/off ratio of 3.16  ×  10^7^. These findings may open a new avenue for the use of low‐dimensional materials to realize wafer‐scale bandgap‐tunable optoelectronics.

## 2 Results and Discussion

### 2.1 Tunable Bandgap of MoS_2_/PbS Heterostructures

To realize bandgap‐tunable heterostructures, it is critical to overcome the limitations of conventional vertical heterostructures. Conventional CVD‐grown vertical MoS_2_/PbS heterostructures typically form a Type‐I straddling band alignment, where the bandgap is inherently fixed by the narrow‐gap semiconductor (PbS) and restricted by weak van der Waals (vdW) coupling. To achieve broad tunability, we propose engineering the interface to transition from a Type‐I to a Type‐II staggered band alignment, which facilitates efficient charge separation and lowers the effective bandgap.

**FIGURE 1:**
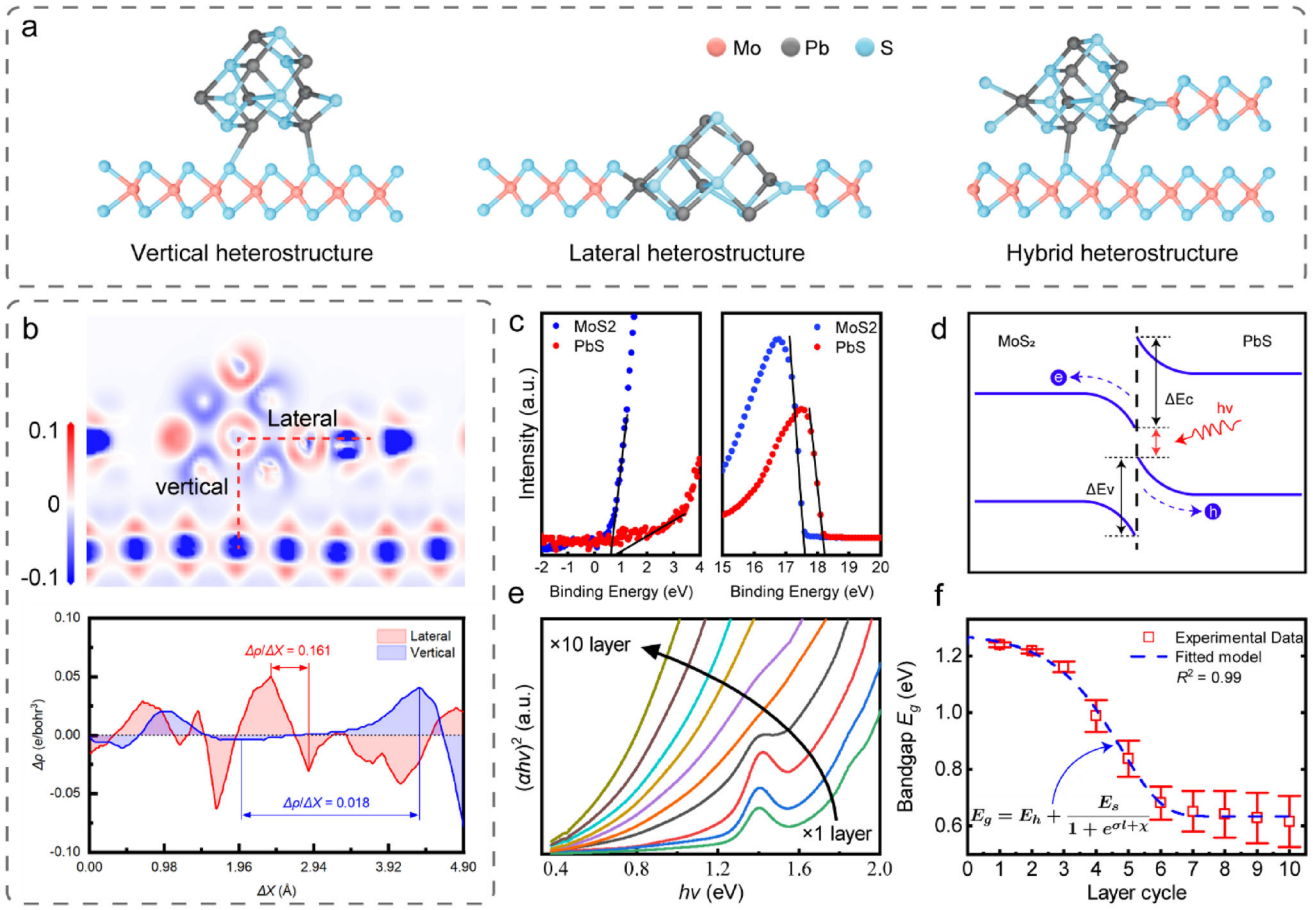
Tunable bandgap of MoS_2_/PbS superlattices. (a) Architectures of vertical, lateral, and hybrid heterojunction (from left to right). (b) The deformation charge density distribution of the hybrid heterojunction (top), and extracted deformation charge density along lateral and vertical directions (bottom) in the distribution. (c) UPS spectra showing the valence band maximum (left) and work function (right) of MoS_2_ and PbS. MoS_2_ presents a work function of 3.86 eV and a *E_VBM_* of 0.59 eV. The PbS showed a work function of 3.04 eV and a *E_VBM_* of 0.68 eV. (d) Band alignment schematic of lateral MoS_2_/PbS heterojunction. Under strong IEF, the charge transfer effect is much more efficient and the band bent. (e) Tauc plot of MoS_2_/PbS heterostructures with 1–10 coating layers, indicating the bandgap (the *hv*‐intercept obtained by extrapolating the linear region of the plot to the *hv*‐axis). (f) The experimentally measured bandgap shows excellent agreement with the theoretical model. The bandgap decreases gradually from 1.24 to 0.61 eV as the number of coating layer increases (n = 3).

To validate this mechanism, we investigated the band structures of three heterojunction architectures—vertical, lateral, and hybrid—via ab initio calculations (see Text S1 and Figure S1). Vertical heterojunctions are formed by stacking MoS_2_ and PbS layers, lateral heterojunctions are constructed through in‐plane bonding of MoS_2_ and PbS, and hybrid architectures incorporate both lateral heterojunctions and vertical heterostructures (Figure 1a). Our calculations confirm that the heterojunction architecture fundamentally dictates the band alignment and the resulting bandgap. The vertical heterojunctions, governed by weak vdW interactions, exhibit a relatively large bandgap of 0.9 eV. In strong contrast, the lateral heterojunctions, formed through in‐plane bonding, allow for significant renormalization of the electronic structure, reducing the theoretical bandgap to ∼0 eV. The hybrid architecture, which incorporates both vertical stacking and lateral bonding, exhibits an intermediate bandgap of 0.21 eV. The deformation charge density (Δ*ρ*) of the hybrid architecture was further calculated to elucidate the interfacial charge distribution of its vertical and lateral interfaces (Figure 1b top). As the spatial gradient of the deformation charge density (Δ*ρ*/Δ*X*) reflects the strength of IEF forces [39], a cross‐sectional deformation charge density distribution map and corresponding lateral/vertical data profiles were extracted (Figure 1b bottom). At the lateral MoS_2_/PbS interface, an increased electron density (0.08 e/bohr^3^) with considerably reduced spatial separation (0.496 Å) is observed. This interface exhibits an interaction strength approximately an order of magnitude greater than that of vertical heterojunctions (0.044 e/bohr^3^ in 2.335 Å). This result implies markedly higher electron transfer efficiency across shorter spatial distances at lateral interfaces, in which carrier injection is significantly enhanced compared with the vertical MoS_2_/PbS configuration, and the dramatic bandgap reduction in lateral and hybrid structures is driven by the enhanced IEF, which promotes the formation of a Type‐II alignment with a smaller effective bandgap.

To experimentally quantify the band edges, we performed the UV photoelectron spectroscopy (UPS) for the Fermi level (*E_F_*) and Valence Band Maximum (*E_VBM_*) measurements on the solution‐processed MoS_2_ and PbS QDs film (Figure 1c). The MoS_2_ film exhibited a work function (Φ) of 3.86 eV and a *E_VBM_* of 0.59 eV. The PbS quantum dots showed a work function of 3.04 eV and a *E_VBM_* of 0.68 eV. To determine the Conduction Band Minimum (*E_CBM_*), we utilized the optical bandgap (*E_g_*) derived from the Tauc plots of the absorption spectra [40, 41] (Figure S2). Using the equation *E_CBM_* = *E_VBM_*  + *E_g_*, we calculated the conduction band positions relative to the vacuum level. For the MoS_2_, the Tauc plot indicates an optical bandgap of 1.34 eV. By considering these values, we plot the band diagram of the heterostructures (Figure 1d; Figure S3). The difference in work functions (ΔΦ  =  0.82 eV) confirms that electrons transfer from PbS to MoS_2_ to align the Fermi levels. This equilibration induces a quantifiable shift of conduction and valence band edges shift in Δ*E_c_* = 0.75 eV, Δ*E_v_* = 0.73 eV, respectively and confirming a Type‐II staggered configuration which is distinct from the traditional MoS_2_/PbS vertical heterostructures (Text S2). With this band alignment, the benefits of the lateral heterojunction are amplified by higher junction densities. Specifically, the abundant intersheet gaps in solution‐processed MoS_2_ induce a stronger built‐in electric field and pronounced band bending. These factors collectively narrow the effective bandgap at the interface, allowing for direct electron transitions from the PbS valence band to the MoS_2_ conduction band. Consequently, this alignment facilitates interlayer photon absorption and efficient carrier separation.

To investigate the effects of solution processing parameters on the MoS_2_/PbS heterostructures, MoS_2_ and PbS films were spin‐coated, using ink solutions of different concentrations and varying rotational speeds separately. Specifically, higher MoS_2_ ink concentrations or lower spin‐coating speeds decrease the MoS_2_ microsheet spacing, thereby suppressing PbS QD intercalation. Conversely, lower MoS_2_ ink concentrations or higher spin‐coating speeds produce excessive interlayer distances. Excessively low solution concentrations undermine the utilization of the high electron transfer efficiency of MoS_2_ due to excessive inter‐sheet spacing. Therefore, by regulating the ink concentrations and rotational speeds, we can modulate PbS QD adsorption on MoS_2_ edges, which is the density of lateral heterojunctions. Hence, MoS_2_/PbS heterostructures with tunable vertical and lateral heterojunctions can be fabricated using this facile yet robust spin‐coating approach. In addition, layer‐by‐layer spin‐coating can further regulate vertical and lateral heterojunctions. In summary, during solution processing, the heterostructure configuration can be engineered through (i) the ink solution concentration, (ii) the spin‐coating speed, or (iii) the number of spin‐coating cycles.

As predetermined ink solution concentration and rotational speed enable reliable formation of thin films, a mathematical model was established to determine over what range the bandgap could theoretically be tailored by changing the number of spin‐coating cycles, which also represents the number of heterostructure layers. By treating MoS_2_ and PbS heterostructures as grand canonical ensembles, we developed a quantitative relationship between the bandgap and the number of layers in heterostructure materials through grand partition function calculations [42] (see Methods—Bandgap model of MoS_2_/PbS superlattices).

$$\;E_{g}=E_{h}+\frac{E_{s}}{1+e^{\left(\sigma l+\chi \right)}}$$

where *E_g_* denotes the overall effective bandgap of the superlattice, *E_h_* represents the sum of the bandgaps of the constituents, *E_s_* is quantitatively related to the bandgap reduction effect in lateral heterojunctions, σ and χ are parameters that depend on the Fermi level of the material and temperature, and *l* signifies the number of heterostructure layers. Overall, the total bandgap modulation derives from two synergistic contributions. The primary term *E_h_*, governed by the bandgap alignment of the heterostructure constituents, establishes the fundamental electronic landscape. The second term corresponds to the bandgap reduction arising from intralayer exciton formation within the structure. This equation can also be used to determine the number of layers that need to be deposited to achieve a specific bandgap.

Using MoS_2_ monolayer and PbS QD ink materials, we assembled MoS_2_/PbS heterostructures through a spin‐coating approach (Text S3). Importantly, the MoS_2_ film fabricated through the solution‐processing method is composed of microscale MoS_2_ nanosheets, and the intersheet gaps facilitate the subsequent infiltration of PbS QDs and formation of lateral heterojunctions. Heterostructures with various coating layer cycles (*l* = 1–10) were spin‐coated and assessed by UV–Vis–NIR spectroscopy (200–3300 nm) to measure layer‐dependent bandgap tunability (Figure S4). The absorbance spectra of MoS_2_/PbS heterostructures exhibit strong absorption in the visible light region, indicating their semiconducting nature and potential applications as wide bandgap photoabsorbers. The corresponding direct *E_g_* (see Text S4 and Figure S5 for information of indirect bandgap) was calculated by the Tauc method [43] (Figure 1e). The average *E_g_* of MoS_2_/PbS gradually decreases from 1.24 to 0.61 eV as the number of layer deposition cycles *l* increases from 1 to 10. The fitted results demonstrate a characteristic S‐shaped decay (*R^2^* = 0.99, Figure 1f) with the theoretical model, validating the proposed bandgap modulation mechanism, which holds significant theoretical potential for extension to other 2D/0D systems (see Text S5).

### 2.2 Solution Processing for Wafer‐Scale Heterostructures

To characterize the spin‐coating ink solutions and fabricate wafer‐scale MoS_2_/PbS superlattices, we prepared MoS_2_ monolayers dispersed in isopropanol and PbS QDs dispersed in n‐Octane [19]. As aggregation readily occurs upon precipitation of low‐dimensional materials, thereby compromising sample integrity, the concentrations of ink solutions were determined through UV–Vis–NIR absorbance measurements according to the Lambert‐Beer law that the absorbance of an ink solution is directly proportional to its concentration. And the MoS_2_ and PbS ink solutions used in this paper were determined by the intensities of the characteristic peaks in the absorption spectra. Standard ink solution with absorption intensity of 0.68@442 nm for MoS_2_ and 1.9@242 nm for PbS was used throughout the study (Figure S6).

**FIGURE 2:**
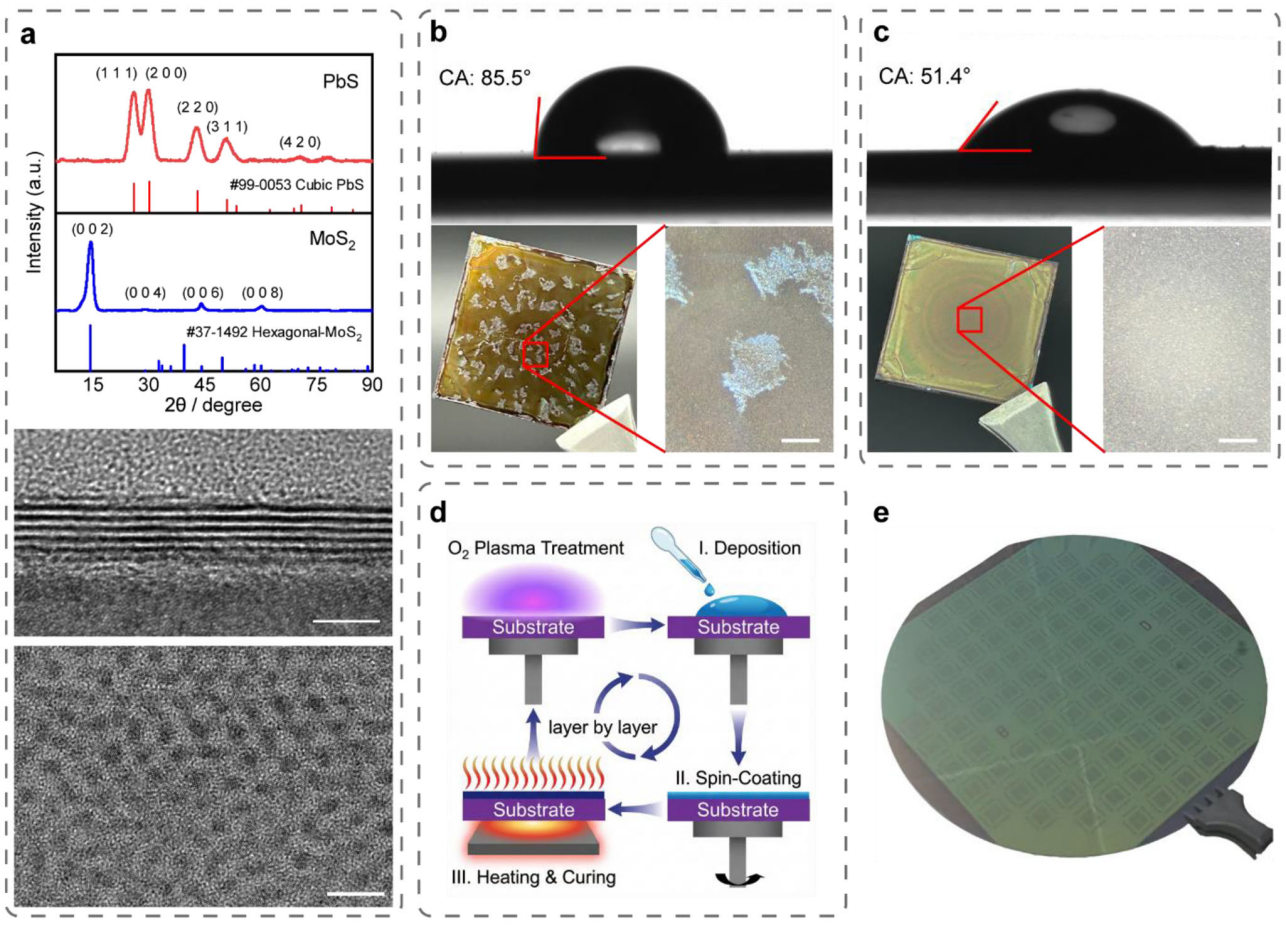
Solution processing for wafer‐scale superlattices. (a) XRD pattern (top), cross‐section HRTEM (middle) of the solution‐processed MoS_2_, and HRTEM (bottom) of the PbS film. The obtained XRD spectrum were compared with the standard reference database [44] confirming the high crystallinity and purity of the obtained MoS_2_ and PbS QDs thin films. The XRD results indicate the spin‐coated MoS_2_ demonstrates strong diffraction peaks at the (0,0,2), (0,0,4), (0,0,6), and (0,0,8) crystal planes with well‐aligned 2D stacking orientation. Cross‐sectional HRTEM of MoS_2_ and HRTEM of PbS quantum dots indicate high sample quality (b,c) The contact angle (top) and heterostructure sample (*l* = 2, bottom) of MoS_2_ before and after O_2_ plasma treatment. The sample without O_2_ plasma exhibiting inferior quality, while the sample after plasma exhibiting significant improvement. The contact angle of O_2_ plasma‐treated spin‐coated MoS_2_ showing suitable for spin‐coating. (d) Layer‐by‐layer assembly method to achieve heterostructures with the required number of layers. (e) A 4‐inch SiO_2_/Si wafer fabricated with transistor arrays using the plasma‐improved solution‐processing method.

The phase purity of the spin‐coated thin film samples was characterized using X‐ray diffraction (XRD), and the obtained spectra were compared with the standard reference database (ICDD PDF database [44]) to confirm the high crystallinity and purity of the obtained MoS_2_ and PbS QD thin films. The XRD data (Figure 2a top) indicate that the spin‐coated MoS_2_ has strong diffraction peaks corresponding to the (0,0,2), (0,0,4), (0,0,6), and (0,0,8) crystal planes, suggesting a well‐aligned 2D stacking orientation between MoS_2_ nanosheets. The XRD results of PbS QDs show that the positions and intensities of diffraction peaks on (1,1,1), (2,0,0), (2,2,0), and other crystal planes are similar to #99‐0053 [44], indicating a typical cubic crystal configuration. The XRD results further demonstrated the high structural integrity and phase purity of ink solutions.

The MoS_2_ and PbS thin films were further investigated by high‐resolution transmission electron microscopy (HRTEM, Figure 2a, middle and bottom). Cross‐sectional HRTEM (Figure S7) of MoS_2_ demonstrates extensive layer‐to‐layer contact among the dangling‐bond‐free MoS_2_ nanosheets, which closely resemble the natural vdW interfaces found between the atomic layers of MoS_2_ crystals. High‐quality, dangling‐bond‐free contact with pinning‐free vdW interfaces optimizes charge transport between individual nanosheets in the thin film, which is crucial for achieving superior electrical performance of optoelectronics. HRTEM of the PbS QDs reveals monodispersed PbS QDs with a particle size of ∼3 nm that self‐assembled into a hexagonal arrangement. These results indicate that the prepared spin‐coating solution ink can be used to fabricate MoS_2_/PbS superlattices.

However, the heterostructure film exhibited pronounced cracking and non‐uniformity (Figure 2b). To improve solution‐processing methods for wafer‐scale heterostructure fabrication, we investigate the interfacial energies via optical contact angle measurement. We found that significant surface energy disparities exist between MoS_2_ and PbS films. The MoS_2_ film exhibited a high contact angle of 85.5°, indicating poor wettability and consequently compromising the quality of the spin‐coated PbS layer, which is susceptible to cracking during the drying process. We therefore treated the spin‐coated MoS_2_ film with oxygen plasma treatment under different processing parameters during the solution processing to modulate their interfacial energies. (Figures S8, S9 and Table S1). After the plasma optimization process (Text S6), the condition of 50 W for 15 s was applied as the optimal condition owing to the achieved contact angle in the ideal range of 45°–60°, which corresponds to favorable wettability and enhanced film uniformity [45] (Figure 2c). To fabricate multilayer heterostructures, the spin‐coating procedures for the MoS_2_ nanosheets and PbS QDs were sequentially iteratively repeated to achieve the target number of heterostructure layers (Figure 2d). Finally, the assembled material was thermally annealed at 400 °C for 1 h in a high‐vacuum furnace (base pressure < 1 × 10^−6^ Pa) to optimize its structural and electronic properties, including its crystallinity and charge transport efficiency.

**FIGURE 3:**
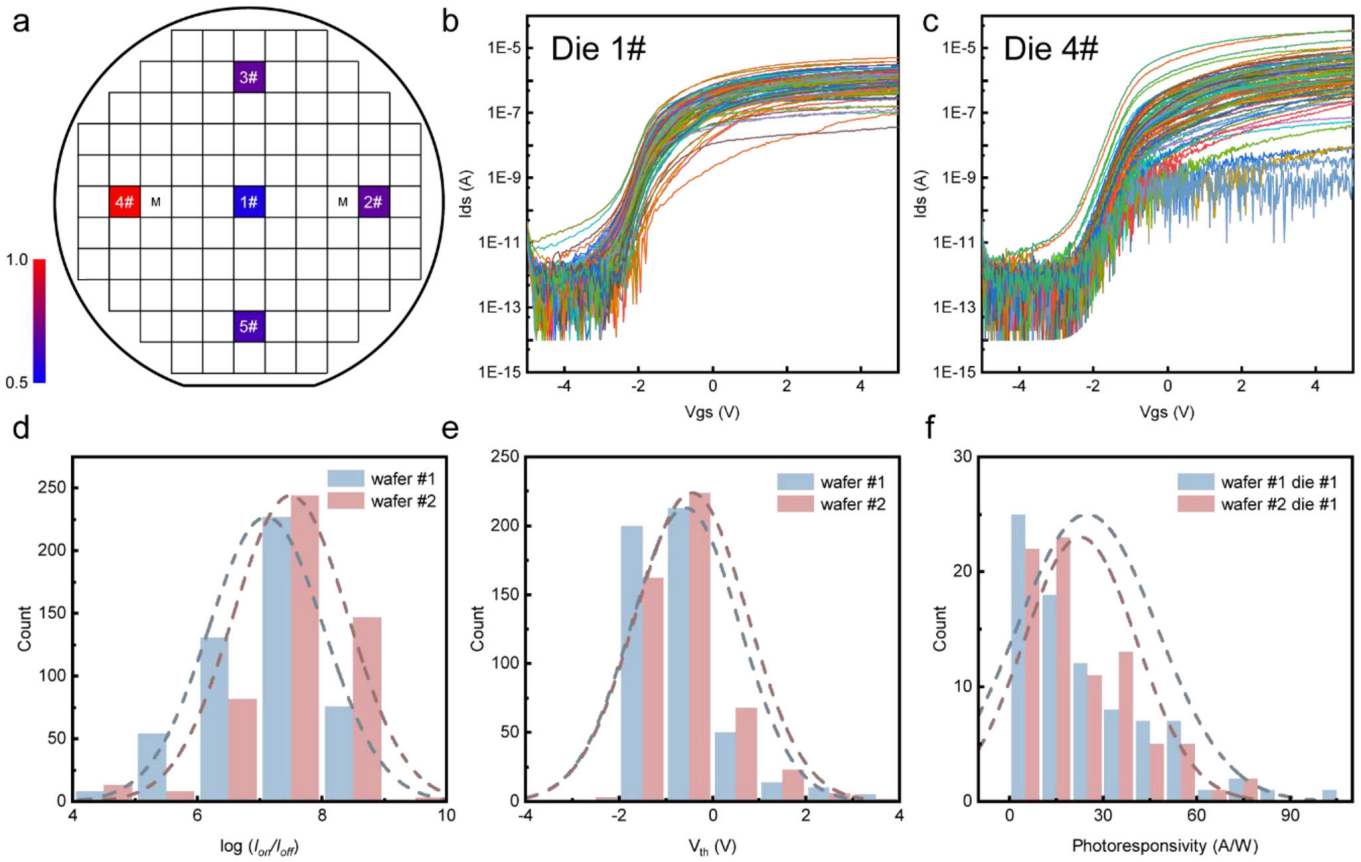
Statistical metrics for wafer‐scale heterostructures. (a) Schematic of the five‐point sampling locations on the wafer, labeled with their respective die numbers. The color shading represents the standard deviation of the on/off ratio, with blue shades indicating lower deviation and red shades indicating higher deviation. Among the tested dies in wafer 1#, die 1#, 2#, 3#, and 5# exhibit relatively low deviations, whereas die 4# shows a higher deviation. (b,c) Transfer characteristic curves of 100 transistors from die 1# and die 4#. The curves from die 1# are relatively concentrated, whereas those from die 4# exhibit greater dispersion. This indicates that the center die (1#) has superior electrical uniformity compared to the edge die (4#). (d,e) Distribution of switching ratios and threshold voltages on wafer 1# and 2#. The threshold voltage was extracted using the constant‐current method, with an on‐current of 0.05 µA. (f) Photoresponsivity measured at V_ds_ = 1 V under 635 nm illumination (20 µW).

To demonstrate the wafer‐scale uniformity of the plasma‐enhanced fabricating method, traditional 4‐inch 300 nm SiO_2_/Si wafer were used to fabricate MoS_2_/PbS transistor arrays with a single coating layer (Figure 2e, see Subsection 2.3 “Phototransistor characteristics” for fabrication details). A total of 1000 devices from two 4‐inch wafers (wafer 1#, 2#) were characterized. This was based on 100 devices per die, selected from five measurement points on each wafer, to ensure representative statistical analysis (see Figure 3a for wafer 1#). Statistical analysis of the key performance figures of merit – on/off switching ratio, threshold voltage (V_th_), and responsivity (R) were performed (Figure 3b–f; Figures S10–S14). The devices exhibited a high average on/off ratio of 3.16  ×  10^7^. The histogram of the logarithmic on/off ratios fits a Gaussian distribution with a tight σ  =  0.92, demonstrating that the plasma treatment effectively minimizes surface energy disparities, thereby preventing the formation of large cracks or defects that typically degrade the off‐state current in solution‐processed films. We also observed a mean threshold voltage of 0.42 V with a standard deviation of ± 1.62 V and responsivity of 27.14 A/W with a standard deviation of ± 22.15 A/W under 635 nm illumination (Figure S14, V_ds_ = 1 V, laser power 20 µW). Yield was defined based on the ±3σ window of the on/off ratio distribution, giving a final value of 97% (Figure S11a). These large‐scale statistical results confirm that our method yields highly reproducible devices with performance metrics comparable to, or partly exceeding, those of exfoliated or CVD‐grown counterparts, fully supporting the “wafer‐scale” capability highlighted in our title.

### 2.3 Phototransistor Characteristics

**FIGURE 4:**
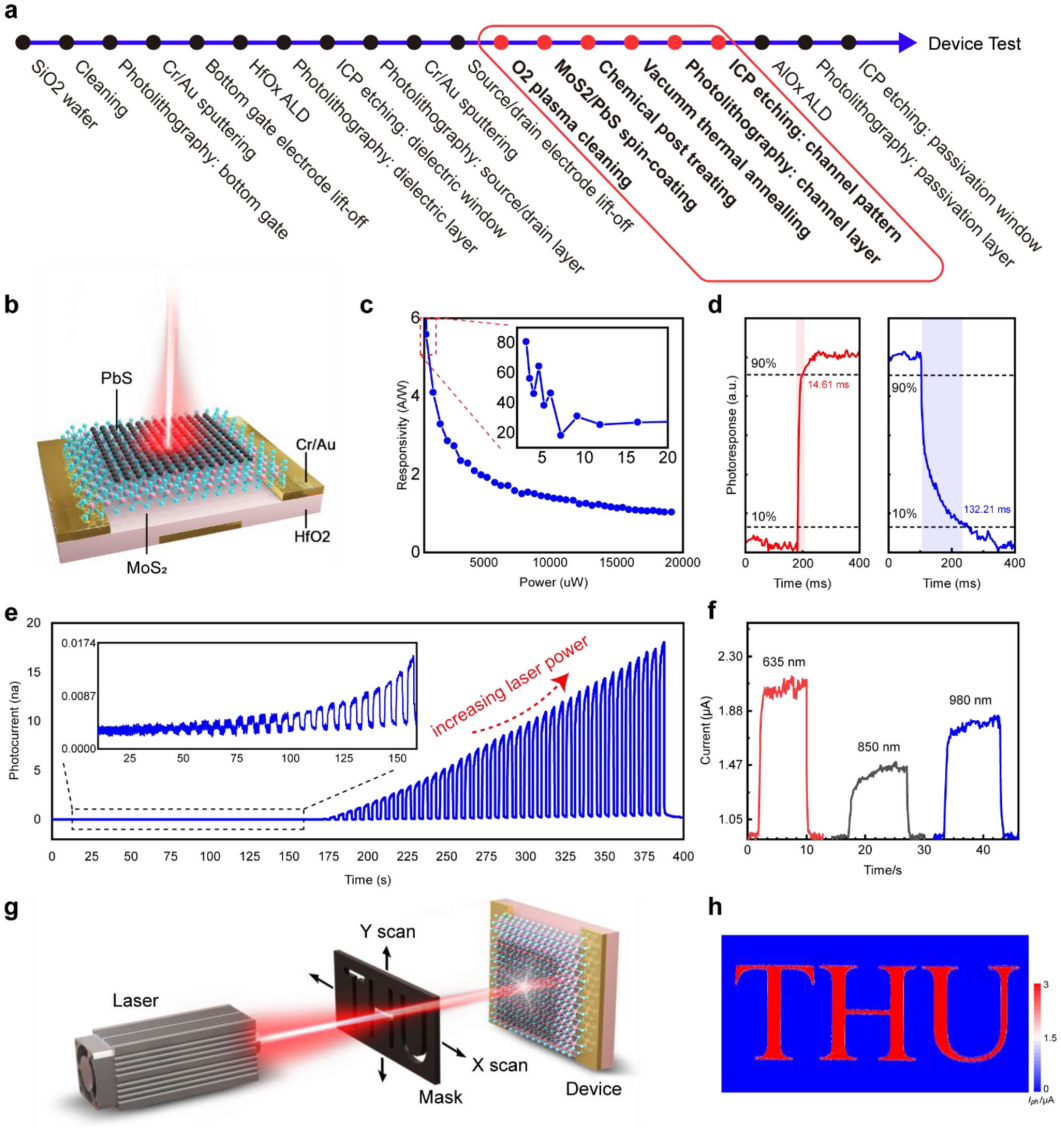
Fabrication and characterization of MoS_2_/PbS phototransistor. (a) Phototransistors fabrication process, the red box shows the solution‐processed heterostructures channel preparation, which is compatible with standard CMOS technology. (b) The schematic structure of the phototransistor using MoS_2_/PbS heterostructures with a bottom‐gate configuration of MOSFET. (c) Responsivity of the phototransistor with illumination power from 3 µW to 19 mW. (d) The rise (τ_*rise*_) and fall (τ_*fall*_) times of the device. The measured response times are in the millisecond range. (e) Photocurrent variation (V_ds_ = 1 V, V_gs_ = −2 V). As time increases, the laser gradually increases the power and switches on and off repeatedly. The relationship between photocurrent and time is recorded. As the power increases, the photocurrent remains stable and shows good linearity. (f) Photocurrent under 635, 850, and 980 nm laser irradiation, demonstrating its application in the near‐infrared region. (g) Schematic diagram of the imaging test setup. The laser beam is directed through the scanning middle photomask for 2D scan. The optical intensity can be obtained point‐by‐point. (h) “THU” characters obtained from 2D scanning of the photocurrent under 635 nm laser.

To evaluate optoelectronic properties, MoS_2_/PbS heterostructures were fabricated into phototransistor devices. For the phototransistor characterization, a device with one single coating layer (*l* = 1) was chosen, as increasing the number of coating layers degrades gate controllability, which in turn reduces the on/off ratio. The wafer‐scale fabrication process of the phototransistor is compatible with standard complementary metal–oxide–semiconductor (CMOS) technology (Figure 4a, see Text S7 for details description of CMOS compatibility). Structures of the MoS_2_/PbS phototransistor are a bottom‐gate configuration to avoid interference with light reception from the top side. High‐k dielectric (HfO_2_, 7 nm) was used as the dielectric layer, Cr (5 nm)/Au (30 nm) was used as the drain, source, and gate electrodes (Figure 4b). The final device could be packaged into the CLCC48 configuration for further applications without a probe station (Figure S15).

The transfer characteristics were measured under a source–drain voltage of 1 V, with the gate voltage swept from −5 to 5 V. The results demonstrate a gate‐controllable on/off ratio exceeding 1.8 × 10^8^ (Figure S16). The optical performance of the heterostructure phototransistor was assessed using a laser centered at 635 nm (Figure 4c–e). The photoresponse of the solution‐processed phototransistor was systematically measured under controlled illumination conditions. The photocurrent exhibits a wide dynamic range under incident power densities from 3 µW to 19 mW. Considering that the responsivity (*R*) is a function of the incident power, we depict the variation in *R* with the incident power (Figure 4c,e). The responsivity is defined as the ratio of the photocurrent to the incident power, 𝑅 = 𝐼_ph_/𝑃_in_, where 𝐼_ph_ is the difference between the current under illumination (𝐼_illu_) and the dark current (𝐼_dark_) and 𝑃_in_ is the incident power. As the incident power increases, the recombination of photogenerated electron–hole pairs becomes more significant, leading to a notable decrease in *R* and a tendency for the photocurrent to saturate at high power levels. Under an effective incident power of 3.1 µW, *R* reaches a maximum of 80.5 A/W. Based on the typical dark current (*I_dark_*) of 0.5 nA, the specific detectivity (D*) is estimated to be 6.74 × 10^12^ Jones for a device area (*A*) of 20×50 µm^2^. This calculation assumes the noise floor is primarily limited by the shot noise of the dark current ($i_{shot}=\sqrt{2qI_{dark}}\;\approx 1.27\times 10^{-14}$ A/Hz^1/2^_,_ $D^{*}=\;R\sqrt{\left(A\right)}/i_{\mathrm{shot}}$). The rise (τ_*rise*_ =  14.61 ms) and fall (τ_*fall*_ =  132.21 ms) times of the device was measured. To demonstrate near‐infrared (NIR) photodetection functionality, the devices were characterized under illumination from 850 nm and 980 nm lasers (Figure 4f). The photocurrent generated at 980 nm was found to be slightly greater than that at 850 nm. The reduced photocurrent observed at 850 nm is primarily attributed to the spectral mismatch with the absorption profile of the MoS_2_/PbS heterojunction, where this wavelength coincides with an absorption minimum of PbS (Figure S6). For long‐term stability evaluation, the device, protected by a dielectric encapsulation AlO_x_ layer on top, was expected to maintain its performance. To assess this, transfer characteristic curves were tested at weekly intervals. The results showed that the device with AlO_x_ protection exhibited good stability, whereas the one without AlO_x_ protection showed slight degradation over the testing period (Figure S17). Furthermore, Tables S2 summarize all the relevant MoS_2_‐based, PbS‐based, and hybrid photodetector parameters, providing a comprehensive overview of our solution‐processed MoS_2_/PbS optoelectronic properties.

Optical imaging measurements were conducted to evaluate the imaging capabilities of the devices. The optical imaging system comprises a laser, a photomask, and the phototransistor (Figure 4g). A 3D‐printed opaque photomask featuring a hollowed‐out “THU” pattern (with a minimum line width size of 1 mm in the character “U”) was chosen as the imaging target. The photomask undergoes 2D scanning during the imaging process. Laser beams of 635, 850, and 980 nm were used, passing through the hollowed section of the plate, reaching the detector and forming images depicting the characters “THU” (see Figure 4h for 635 nm, and Figure S18 for 850, 980 nm). Images show promising clarity and precision, with a resolution exceeding 0.1 mm, constrained by the stepping accuracy of the motion system and the configuration of the laser source projection. This indicates that the device is suitable for application in the NIR spectral band and provides valuable guidance for future multispectral designs (see Text S8 for details).

## 3 Conclusion

In this work, we present advancements in wafer‐scale optoelectronic sensors through the design and fabrication of MoS_2_/PbS heterostructures with lateral heterojunctions using the plasma‐improved solution‐processing method. Lateral heterojunctions formed between the MoS_2_ nanosheets and PbS QDs in the heterostructures result in tunable bandgap characteristics from 1.24 to 0.61 eV. Furthermore, we propose a bandgap modulation model that exhibits promising predictive accuracy, achieving a coefficient of determination (*R^2^*) of 0.99. By utilizing these solution‐processed heterostructures, phototransistors with remarkable features, including a maximum responsivity of 88 A/W, specific detectivity of 4.77  ×  10^12^ Jones, and a typical on/off ratio of 3.16  ×  10^7^ and fabrication scaling from traditional micro‐scale devices to 4‐inch wafer‐scale devices with near‐ideal yields of 97% were developed, demonstrating promising potential for diverse sensing applications. These experimental results indicate that the wafer‐scale solution‐processing method for tailoring heterostructures may pave the way toward next‐generation optoelectronic devices.

## 4 Experimental Section

### 4.1 Synthesis of PbS QDs and MoS_2_ Nanosheets

Synthesis of PbS QDs was carried out using standard Schlenk techniques. 2 mmol PbO, 8 mmol 1‐Octadecene (ODE), and 4 mmol oleic acid were added to a three‐necked flask and vaccumed overnight at 95 °C to dewater. Then 15 mL ODE was added, and the temperature of the reaction was set to 120 °C. When 120 °C was reached, 1 mmol HMS mixed with 10 mL ODE were quickly injected. The heating was stopped without removing the heating mantel and the reaction was allowed to cool down to room temperature slowly. The reactant nanocrystals were isolated by the addition of acetone and centrifugation, purified by dispersion/precipitation with toluene/acetone, and finally dispersed in n‐Octane. Synthesis of MoS_2_ nanosheets were based on electrochemical molecular intercalation and exfoliation of MoS_2_ crystals. The reaction products are cleaned and dispersed in IPA. Concentrations of PbS and MoS_2_ dispersion are then determined by absorption spectroscopy.

### 4.2 Phototransistor Fabrication Process

The transistor fabrication process was carried out using specialized equipment for each critical step in a clean room to get high yield. Pattern definition was achieved through photolithography using an MA8 mask aligner (SUSS MicroTec) with NR9‐3000py photoresist (Futurrex) employed for metal layer patterning and AZ GXR601 photoresist (AZ Electronic Materials) for etch region definition. For dielectric deposition, HfO_x_ and AlO_x_ thin films were grown via atomic layer deposition using R200 Advanced (Picosun) and TFS‐200 (Beneq), respectively. Metallization was performed by JCP500 magnetron sputtering (Technol). Plasma surface treatment was performed using an ION 10 system (PVA TePla). Thermal annealing was conducted in AS‐One100 (ANNEALSYS). ICP etching was accomplished through NE‐550H system (ULVAC). This comprehensive process flow ensured precise control over each fabrication step, enabling high‐performance transistor characteristics.

### 4.3 Characterization

TEM Characterizations were carried out using FIB‐SEM (LYRA3, TESCAN.Q.S) and TEM/STEM (JEM‐2100F), XRD (D/max‐2500/PC), UV–Vis–NIR spectroscopy (Cary5000, Agilent). The electrical characterization of the fabricated phototransistors was performed using a CASCADE probe station under atmospheric conditions with a Keysight B1500A parameter analyser. The transistor photoelectric signal test is conducted through an adjustable power laser and a Keithley 2614B source measure unit. The batch testing of wafer‐scale transistors was performed using an automated probe station (TS2000‐SE, MPI) interfaced with a Keysight B1500A parameter analyser.

### 4.4 Bandgap Model of MoS_2_/PbS Superlattices

Assume there are *N_o_* PbS binding sites within the solution‐processed MoS_2_ layer, each capable of accommodating a PbS quantum dot. The incorporation of quantum dots induces a bandgap shift of ε_0_. Treating PbS as both a heat reservoir and a particle reservoir, the embedded PbS quantum dots can be regarded as a system capable of exchanging particles and energy with the reservoir, obeying the grand canonical ensemble. When *N* quantum dots are embedded, the system's bandgap shifts by *N*ε_0_. Let *E_gm_* denote the bandgap of solution‐processed MoS_2_, *E_gp_* the bandgap of PbS quantum dots, and $\bar{N}$ the average number of quantum dots in the system. Consequently, the bandgap of the MoS_2_/PbS heterostructures can be written as

$$E_{g}=E_{gm}+E_{gp}+{\bar{N}}_{\in_{0}}$$

Considering that *N* PbS quantum dots can be distributed among *N*_0_ embedding sites in $C_{N_{0}}^{N}$ distinct configurations, the grand partition function of the system can then be written as

$$\Xi =\sum_{N=0}^{N_{0}}\frac{N_{0}!}{N!\left(N_{0}-N\right)!}e^{-\alpha N-\beta N\in_{0}}$$

where $\alpha =-\frac{\mu }{kT}$ and $\beta =\frac{1}{kT}$. Here, μ represents the chemical potential, *T* denotes the thermodynamic temperature, and *k* is a constant, hence

$$\Xi =\sum_{N=0}^{N_{0}}\frac{N_{o}!}{N!\left(N_{0}-N\right)!}e^{\beta \left(\mu -\in_{0}\right)N}={\left[1+e^{\beta \left(\mu -\in_{0}\right)}\right]}^{N_{0}}$$

The average number of embedded quantum dots in the system becomes

$$\;\bar{N}=-\;\frac{\partial \ln\Xi }{\partial \alpha }=kT\;\frac{\partial \ln\Xi }{\partial \mu }=\frac{N_{0}}{1+e^{-\beta \left(\mu -\in_{0}\right)}}$$

The Fermi level $E_{f}\approx \mu$, as the Fermi level decreases with increasing layer number *l*. The Fermi level can be seen as

$$\;E_{f}=-\eta l+\theta$$

Here, η and θ are constants. Consequently, the heterojunction bandgap can be expressed as

$$\;E_{g}=E_{h}+\frac{E_{s}}{1+e^{\left(\sigma l+\chi \right)}}$$

where, $E_{h}=E_{gm}+E_{gp}$, $E_{s}=N_{0}\in_{0}$, σ=βη, and $\chi =\beta (\varepsilon_{0}-\theta )$

These parameters could be determined through fitting with experimental data.

## Funding

This work was supported in part by grants from the National Natural Science Foundation of China (62173198, 62425306, 62350710211, and 62173200), in part by the Natural Science Foundation of Beijing Municipal under Grant L232027.

## Conflicts of Interest

The authors declare no conflicts of interest.

## Supporting information




**Supporting File**: advs73725‐sup‐0001‐SuppMat.docx.

**Supporting Video**: advs73725‐sup‐0002‐Movie S1.mp4.

## Data Availability

All data needed to evaluate the conclusions in the paper are present in the paper and/or the Supplementary Materials.
