# Supplementary material for: Wafer‐Scale Bandgap‐Tunable MoS2/PbS Phototransistors Enabled by Solution Processing
Source: Adv Sci (Weinh). 2026 Jan 12;13(16):e18844. doi: 10.1002/advs.202518844 (PMC13042418; doi:10.1002/advs.202518844)
Supplement: Supplementary file 1 — Supporting File: advs73725‐sup‐0001‐SuppMat.docx. [file ADVS-13-e18844-s001.docx]

Supporting Information

Wafer-scale bandgap-tunable MoS2/PbS phototransistors enabled by solution processing

Ziheng Tang, Chengqian Cui, Xiaoli Jing, Rui You, Mingjun Zhang, Jing Xu*

Supplementary Text 1. *Ab initio* calculations of vertical, hybrid, and lateral MoS2/PbS heterojunctions

All the calculations are performed in the framework of the density functional theory with the projector augmented plane-wave method, as implemented in the Vienna ab initio simulation package^[1]^. The generalized gradient approximation proposed by Perdew-Burke-Ernzerhof (PBE) is selected for the exchange-correlation potential^[2]^. The cut-off energy for plane wave is set to 500 eV. The energy criterion is set to 10^−5^ eV in iterative solution of the Kohn-Sham equation. All the structures are relaxed until the residual forces on the atoms have declined to less than 0.02 eV/Å. To avoid interlaminar interactions, a vacuum spacing of 20 Å is applied perpendicular to the slab.

Supplementary Text 2. Band alignment of lateral MoS2/PbS heterojunctions

The theoretical calculations reinforce the experimental findings by quantifying the interaction strength and the resulting electronic structure changes. We calculated the deformation charge density to quantify the coupling strength. The lateral interface exhibits an electron density of $0.08 e/bohr^{3}$ with a significantly reduced spatial separation of 0.496 Å. This is approximately double the density and an order of magnitude stronger than the vertical interface, which shows only $0.044 e/bohr^{3}$ at a separation of 2.335 Å. The shift in band edges is further quantified by the dramatic reduction in the computed bandgap across the different architectures. The bandgap decreases from 0.9 eV in the vertical heterojunction to 0.21 eV in the hybrid, and finally to 0 eV in the purely lateral heterojunction. This theoretical collapse to a semi-metallic state (0 eV) serves as quantitative proof that the conduction and valence band edges have shifted significantly to overlap, driven by the strong lateral coupling.

Based on the quantifiable data above, we can now clarify the specific alteration in alignment: (1) Vertical heterostructures formed by conventional transfer rely on weak van der Waals forces. This results in a weak interfacial electric field (IEF) that is insufficient to significantly perturb the intrinsic band positions, leading to a Type-I (straddling) alignment where the narrower gap (PbS) is contained entirely within the wider gap (MoS₂)^[3]^ (**Figure S3a**). (2) Lateral Mechanism (Type-II): In our solution-processed lateral interfaces, the coupling is significantly stronger. The large difference in Fermi levels combined with the short interaction distance (0.496 Å) drives spontaneous electron transfer from PbS to MoS₂. This generates a strong Interfacial Electric Field (IEF) that bends the energy bands—specifically bending the MoS₂ bands downward and the PbS bands upward **(Figure S3c)**. In conclusion, it is this specific lateral-coupling-induced band bending that alters the alignment from Type-I to Type-II (staggered), enabling the efficient charge separation and tunable bandgap (1.24 to 0.61 eV) between VB of PbS and CB of MoS2 which collectively narrow the effective bandgap at the interface. Consequently, this alignment facilitates interlayer photon absorption and efficient carrier separation.

Supplementary Text 3. Spin-coating of MoS2/PbS superlattices

The concentrations of PbS and MoS2 ink solutions were determined by analyzing their absorption spectra, with solution concentrations calibrated using the absorbance intensity at characteristic wavelengths. The solution concentration was carefully regulated via dilution or concentration to meet a predefined reference standard. Standard ink solution with absorption intensity of 0.68@442nm for MoS2 and 1.9@242nm for PbS was used (**Figure S6**). Based on standard ink solutions, multilayer thin films of MoS2 and PbS were fabricated via sequential spin-coating, and absorbance measurements were performed on selected uniform regions of the samples.

Supplementary Text 4. Direct or indirect bandgap of MoS2/PbS heterostructures

While monolayer MoS2 and PbS quantum dots are individually direct bandgap materials, the formation of a MoS2/PbS superlattice--specifically one with a Type-II staggered alignment—introduces the possibility of indirect transitions, particularly those arising from interlayer coupling where the valence band maximum (VBM) and conduction band minimum (CBM) may be separated in momentum space. To empirically verify the nature of the optical transitions, Tauc plots for both direct ($\left( \alpha h\nu\right)^{2}vs.h\nu$) and indirect ($\left( \alpha h\nu\right)^{1/2}vs.h\nu$) transitions were compared. As shown in **Figure S5**, the indirect transition plots of the MoS2/PbS heterostructures is hard to extract bandgap, especially in high-layer count samples (whose extrapolated intersection point with the x-axis may lie on the negative side). In contrast, the direct transition plots (**Figure 1e**) display clear, extended linear regimes, confirming that direct optical transitions dominate the absorption edge in these heterostructures.

Supplementary Text 5. Theoretical potential for extension to other 2D/0D systems

The tunability observed in the solution-processed MoS2/PbS system arises fundamentally from the formation of lateral Type-II heterojunctions. Unlike vertical structures which form Type-I alignments where the bandgap is pinned to the narrow-gap semiconductor (PbS), the lateral interfaces facilitate strong intralayer charge transfer. This creates an interfacial electric field that bends the energy bands, resulting in a tunable "effective" bandgap that decreases as the density of these lateral junctions increases. Extending this to other combinations requires analyzing their specific band edge positions. We theoretically predict that combinations such as MoSe2/PbSe^[4,5]^ would successfully demonstrate similar bandgap tunability. Both MoSe₂ and PbSe have narrower intrinsic bandgaps compared to their sulfide counterparts (Monolayer MoS2 has a bandgap of ~1.8 eV, while monolayer MoSe2 is narrower, typically ~1.5 eV. Accordingly, bulk PbS is ~0.41 eV, whereas PbSe is ~0.27 eV.), and their band offsets are expected to preserve the Type-II staggered alignment essential for charge separation^[6]^. In this scenario, our solution-processing method would not only allow for tunability but would likely shift the operational range into the Deep Infrared (Deep-IR), as the tunable effective gap would be derived from the narrower MoSe₂/PbSe band edges.

However, extending this to wide-bandgap TMDCs like WS₂ presents a theoretical risk. WS₂ has a significantly wider bandgap and different band edge positions compared to MoS₂. If the conduction and valence bands of the PbS QDs lie entirely within the bandgap of WS₂, the system would form a Type-I (straddling) alignment even in a lateral configuration. In a Type-I regime, photogenerated carriers are confined within the narrow-gap material (PbS) rather than separated across the interface. The optical response would become "pinned" to the intrinsic bandgap of the PbS QDs, causing a loss of the layer-dependent tunability we achieved with MoS₂. Therefore, while the fabrication method is transferable, the tunability is strictly contingent on selecting 2D/Nanocrystal pairs that possess appropriate band offsets to form Type-II lateral heterojunctions.

As for the plasma treatment for achieving wafer-scale uniformity, we demonstrated that oxygen plasma treatment effectively tunes the surface energy of the MoS2 film to match the solvent of the PbS ink. Theoretically, this physical surface modification strategy is applicable to other TMDCs like MoSe2 or WS2, as they share similar crystal structures and initial hydrophobic surface properties. We found that bandgap tunability is dominated by "lateral heterojunctions" formed when QDs infiltrate the physical gaps between solution-processed nanosheets. This mechanism relies on the physical morphology of the nanosheet network rather than specific chemical bonding. Therefore, provided other 2D materials can be processed into nanosheet inks with similar inter-sheet spacing, the formation of tunable lateral heterostructures should be geometrically feasible. A major challenge in transferring this method to selenides (e.g., MoSe2, PbSe) is their **chemical stability**. Our optimized plasma condition was specific to MoS2. Selenium-based materials are generally more susceptible to oxidation than sulfides^[7]^. Consequently, the plasma treatment required to improve wettability might inadvertently cause severe lattice damage or over-oxidation in MoSe₂/PbSe, degrading the carrier transport channels. Our success relied on a specific solvent system (Isopropanol for MoS₂ and n-Octane for PbS) that prevented re-dissolution of the underlying layers. Extending to other materials may require different dispersants to stabilize the inks. Finding a solvent combination that is compatible with the new materials while maintaining "orthogonality" (where the second ink does not damage the first layer) presents a significant physicochemical challenge.

While the fabrication method is transferable, substituting materials will introduce distinct trade-offs in device performance, governed by the intrinsic properties of the new constituents and their band alignments. Assuming the fabrication challenges described above can be resolved, we theoretically expect the following trade-offs based on the intrinsic properties of the alternative materials. Replacing MoS2 with MoSe2 or WS2 could theoretically improve mobility, as these materials often exhibit lower effective masses. However, a critical trade-off lies in the lateral interface quality. Our MoS2/PbS system benefits from "dangling-bond-free contact" which optimizes charge transport. Introducing materials with **larger lattice mismatches** (e.g., WS2/PbS) may increase phonon scattering at the lateral heterojunctions, potentially negating the intrinsic mobility benefits of the 2D channel material. Utilizing narrower bandgap materials like PbSe QDs would extend the detection range deeper into the infrared. However, the expected trade-off is a reduction in the device's on/off ratio. Our MoS2/PbS devices achieve a high on/off ratio largely due to the effective suppression of dark current by the MoS2. A narrower bandgap system would likely suffer from higher thermal noise (dark current), potentially necessitating cooling or more complex device architectures to maintain high detectivity.

Supplementary Text 6. The plasma optimization process

Argon (Ar) and oxygen (O2) plasma treatments are widely established techniques for modulating the surface properties of two-dimensional materials. Mechanistically, these methods operate on distinct principles: argon plasma primarily relies on **physical sputtering** and ion bombardment to clean surfaces and introduce defects, whereas oxygen plasma modifies surface energy through chemical functionalization, introducing hydrophilic groups (such as -OH) and removing organic residues. However, for the solution-processing of MoS2, Ar plasma presents significant disadvantages: its dominant physical bombardment mechanism often induces severe lattice damage and uncontrolled sulfur vacancies, which can degrade the material's electronic properties. Therefore, O2 plasma was chosen for surface treatment. While O2 plasma also carries the inherent risk of over-oxidizing and etching the lattice, modulating the power and treatment time effectively could suppress defect formation and results in optimized interfacial properties. Therefore, we chose a relatively low power (50 W), conducted a series of tests under various time, and measured the corresponding contact angle (**Figure S8,9, Table S1**). Given that the ideal contact angle falls within the range of 45–60 degrees^[8]^, we chose 15 seconds for processing balancing optimizing surface energy and reducing material damage. Higher power and more time will significantly reduce surface energy, but this is unnecessary as too low surface energy can lead to PbS film being too thin and the film being more prone to damage during the drying process.

**Supplementary Text 7. CMOS compatible of solution-processed devices**

Transitioning 2D materials into standard CMOS technology presents challenges that extend beyond mere wafer-scale uniformity^[9,10]^, including contact engineering, dielectric compatibility, and achieving high yield on wafer sizes larger than 4 inches. As for contact engineering, while our current devices utilize Cr/Au electrodes, we recognize that Gold (Au) is strictly prohibited in most CMOS front-end and back-end-of-line (BEOL) facilities due to its role as a **deep-level contaminant in silicon**. A practical integration path requires replacing Au with standard CMOS metallization systems such as Titanium Nitride (TiN), Tungsten (W), or Copper (Cu). This necessitates a new study on the work-function matching and chemical stability between these metals and the MoS2/PbS interface to maintain the high performance reported here. Moreover, standard high-volume manufacturing (HVM) does not support "lift-off" patterning due to yield-killing metallic "ears" and particulate residues. Practical integration will require transitioning to subtractive dry etching or Damascene processes. The primary challenge here is achieving high etch selectivity—finding a plasma chemistry that **patterns** the electrodes or the 2D/QD hybrid channel without damaging the sensitive underlying 2D lattice or the QD ligand environment. What’s more, unlike the flat substrates used in this study, a functional CMOS readout chip (ROIC) possesses significant surface topography from metal interconnects and vias. Spin-coating solution-processed materials over such features leads to radial striations and thickness variations (where the film is thinner over peaks and **accumulates** in trenches). This would lead to unacceptable pixel-to-pixel non-uniformity in a large-scale image sensor. Future work must integrate Chemical Mechanical Polishing (CMP) or intermediate planarization layers to ensure a flat landing zone for the MoS2/PbS active layer. As for yield at wafer-scale beyond 4 inches. Scaling to 8-inch or 12-inch wafers introduces fluid dynamics challenges, particularly edge-beading and increased centrifugal gradients. While our surface energy modulation (plasma treatment) provides a physics-based solution for wettability, moving beyond 4 inches may necessitate a transition from spin-coating to slot-die coating or spray-coating, which are more material-efficient and capable of maintaining uniformity over larger diameters. By identifying these specific challenges, we demonstrate that our current work serves as the foundational "material-system proof," providing the uniform active layer onto which these standard CMOS industrial processes can be optimized.

**Supplementary Text 8. Future multispectral detection designs**

As well as impact on future multispectral detection designs. Our findings suggest a paradigm shift from physical assembly to **material-level engineering** for multispectral applications. Traditional multispectral cameras often rely on external filters or complex integration of different sensor materials^[9]^ (e.g., silicon for visible, InGaAs for IR). Our solution-processing method allows the bandgap to be tuned simply by controlling the number of spin-coating layer cycles. This means that the spectral response range can be engineered directly during the film fabrication process. This approach establishes a pathway for wafer-scale fabrication where different pixels or regions on a single chip can be processed with different layer cycles. Consequently, a single monolithic sensor array could possess distinct regions sensitive to different spectral bands (e.g., visible vs. NIR) without the lattice mismatch issues typically associated with hetero-integration. By tuning the bandgap to match the specific target wavelength (e.g., 980 nm or 1550 nm), the device can minimize thermal noise associated with unnecessarily small bandgaps, thereby optimizing the specific detectivity for the desired spectral band. We believe these capabilities demonstrate that our MoS2/PbS superlattices are not only effective in the NIR range but also offer a scalable, solution-processed platform for next-generation, filter-free multispectral imaging systems.

**Figure S1 | *Ab initio* band gap calculations of vertical (a,d), hybrid (b,e), and lateral (c,f) MoS2/PbS heterojunctions**


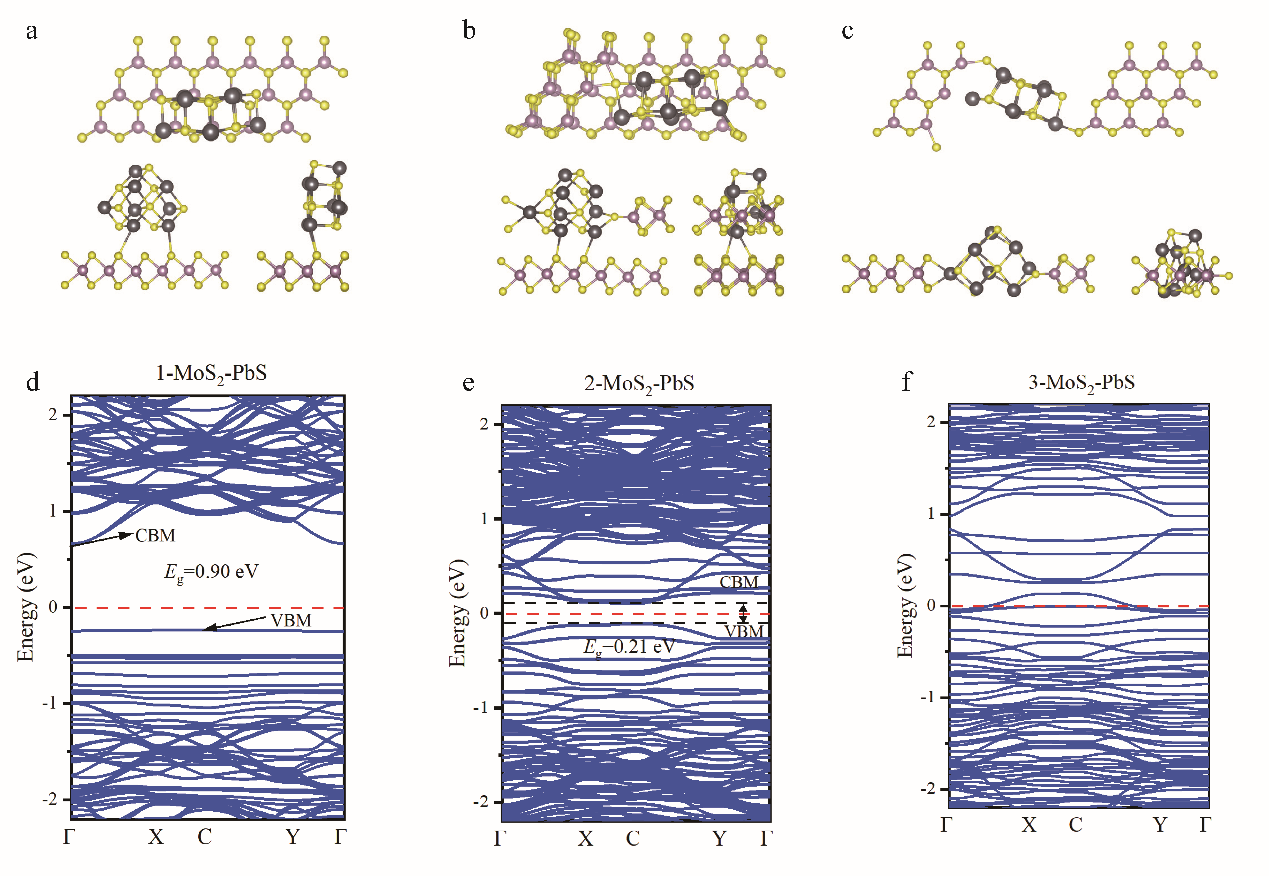


Figure S2. Tauc plot of MoS2 (a) and PbS (b) indicating bandgap of 1.34 and 1.36 eV respectively.

**
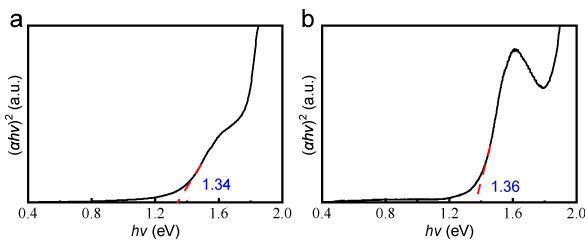
**

Figure S3. Band alignment of lateral MoS2/PbS heterojunctions a, The conventional vertical MoS₂/PbS heterostructure exhibits a Type-I band alignment^[3]^, where the vdW gap at the interface forms a barrier that impedes carrier transport. b, Band diagram obtained from UPS and UV-Vis-NIR absorption spectra, indicating that the solution-processed materials exhibit a Type-II alignment. c, Schematic illustration of the band alignment in lateral MoS2/PbS, the strong carrier transport capability of the lateral heterostructure results in significant band bending and effectively reducing the effective optical band gap.


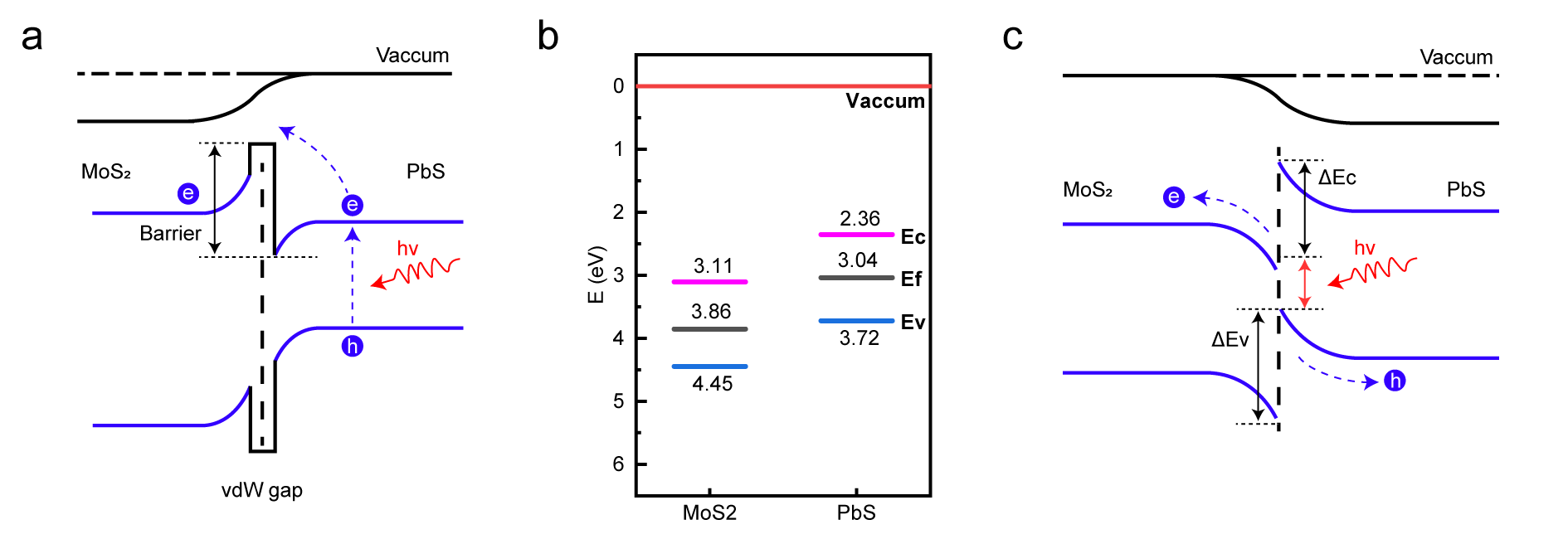


**Figure S4 | Absorbance spectra of MoS2/PbS heterostructures ( *l* = 1-10 )**


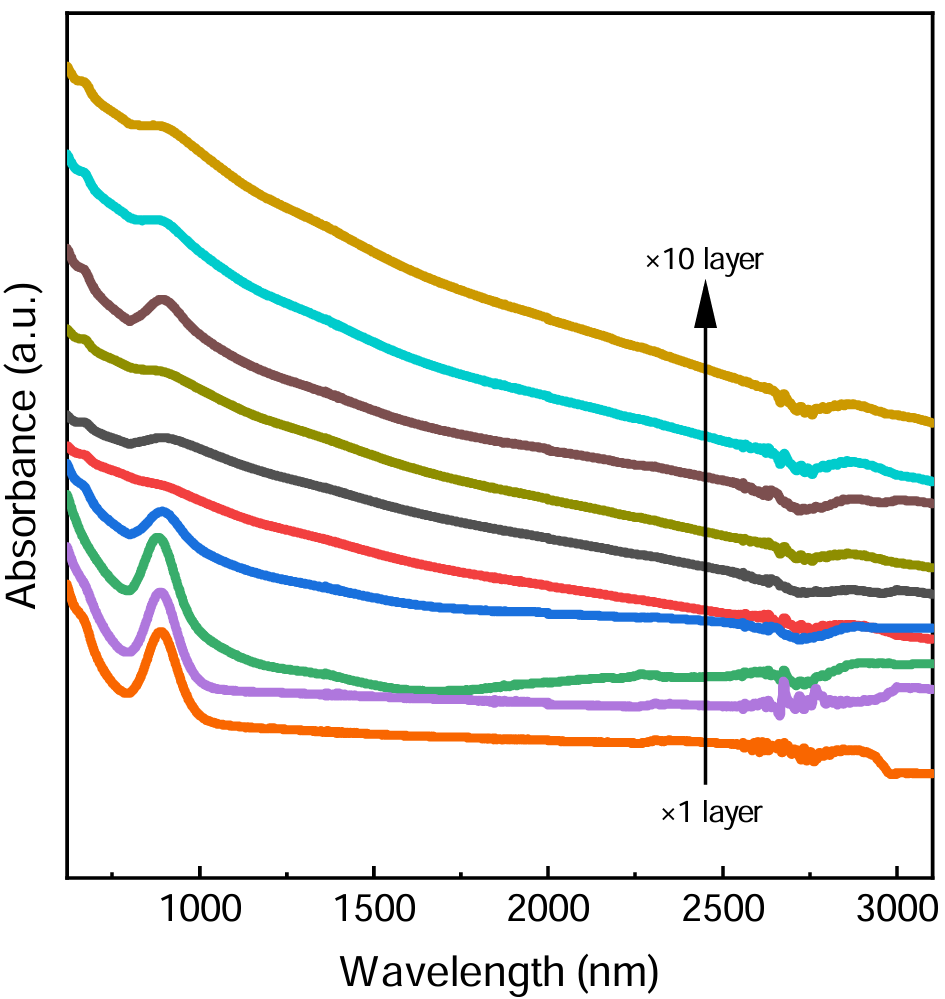


**Figure S5 | The Tauc plot of indirect bandgap** $\left( \boldsymbol{\alpha}\boldsymbol{h}\boldsymbol{\nu} \right)^{\mathbf{1}\mathbf{/}\mathbf{2}}\boldsymbol{- h}\boldsymbol{\nu}$ confirming that direct optical transitions dominate the absorption edge in these heterostructures, as the indirect transition plots of the MoS2/PbS heterostructures is hard to extract bandgap, especially in high-layer count samples (whose extrapolated intersection point with the x-axis may lie on the negative side)


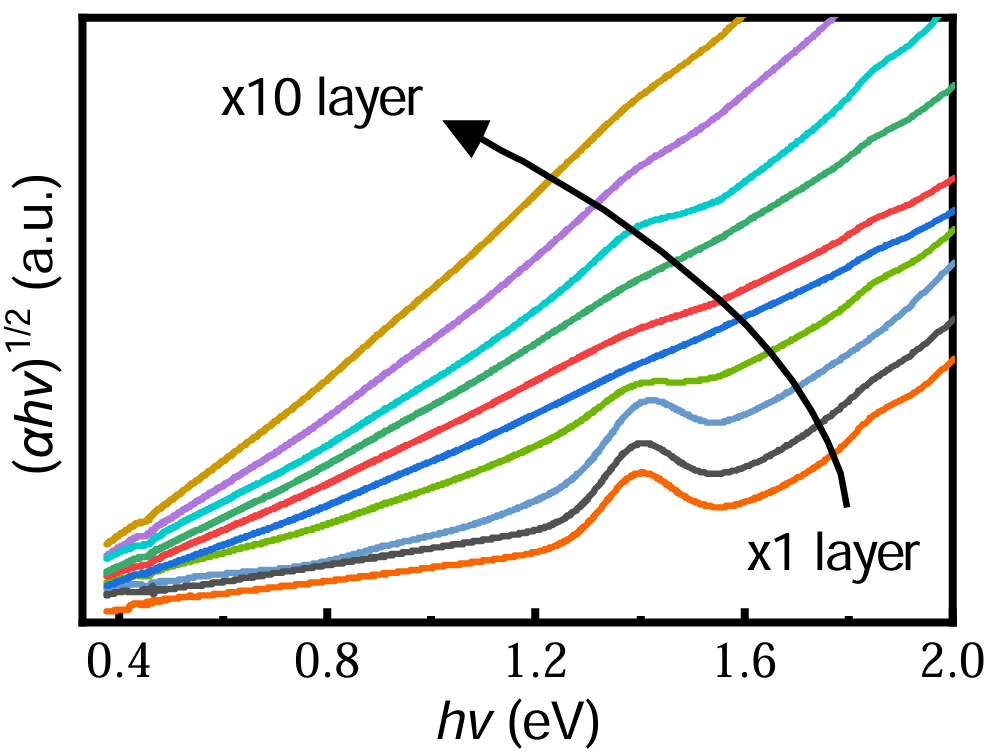


**Figure S6 | UV-Vis absorbance of solution inks of PbS and MoS2**


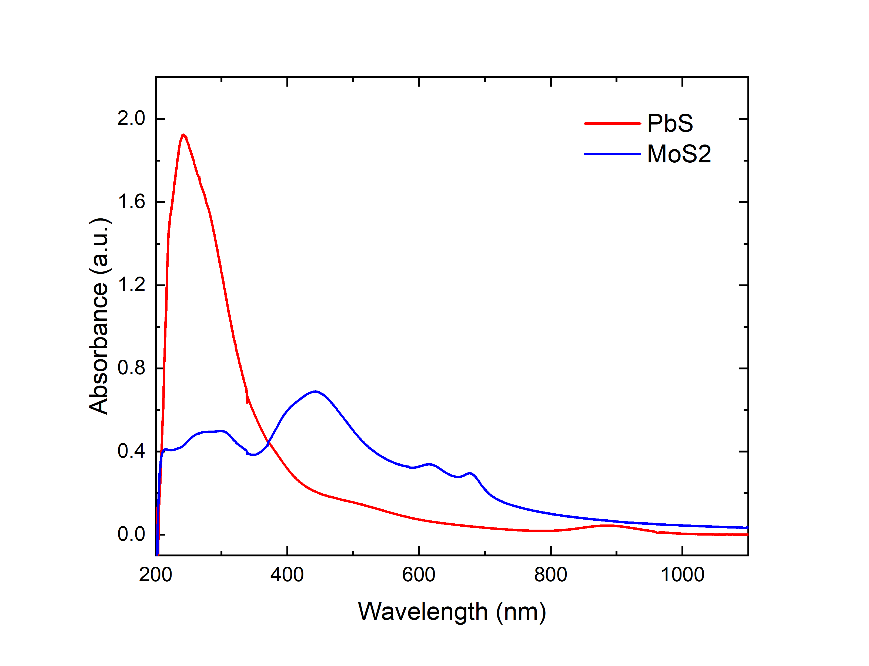


**Figure S7 | The FIB procedure for cross-sectional HRTEM of the MoS2** A FIB lift-out procedure was employed to prepare the HRTEM sample. Initially, a protective layer of Cr **(a)** and Pt **(b)** was deposited on the sample surface to prevent damage during processing. Subsequently, two large trenches were milled on both sides of the target region using the ion beam **(c**, "bulk-out"**)**, leaving the lamella attached to the bulk material by only a small connection **(d,** "U-cut"**)**. The nanomanipulator was then engaged to extract the lamella **(e,** "Lift-out"**)**, which was subsequently transferred and welded onto a copper grid **(f,g)**. Finally, the lamella was thinned to approximately 100 nm **(h)** using a low-energy ion beam to ensure electron transparency for TEM observation **(i)**.


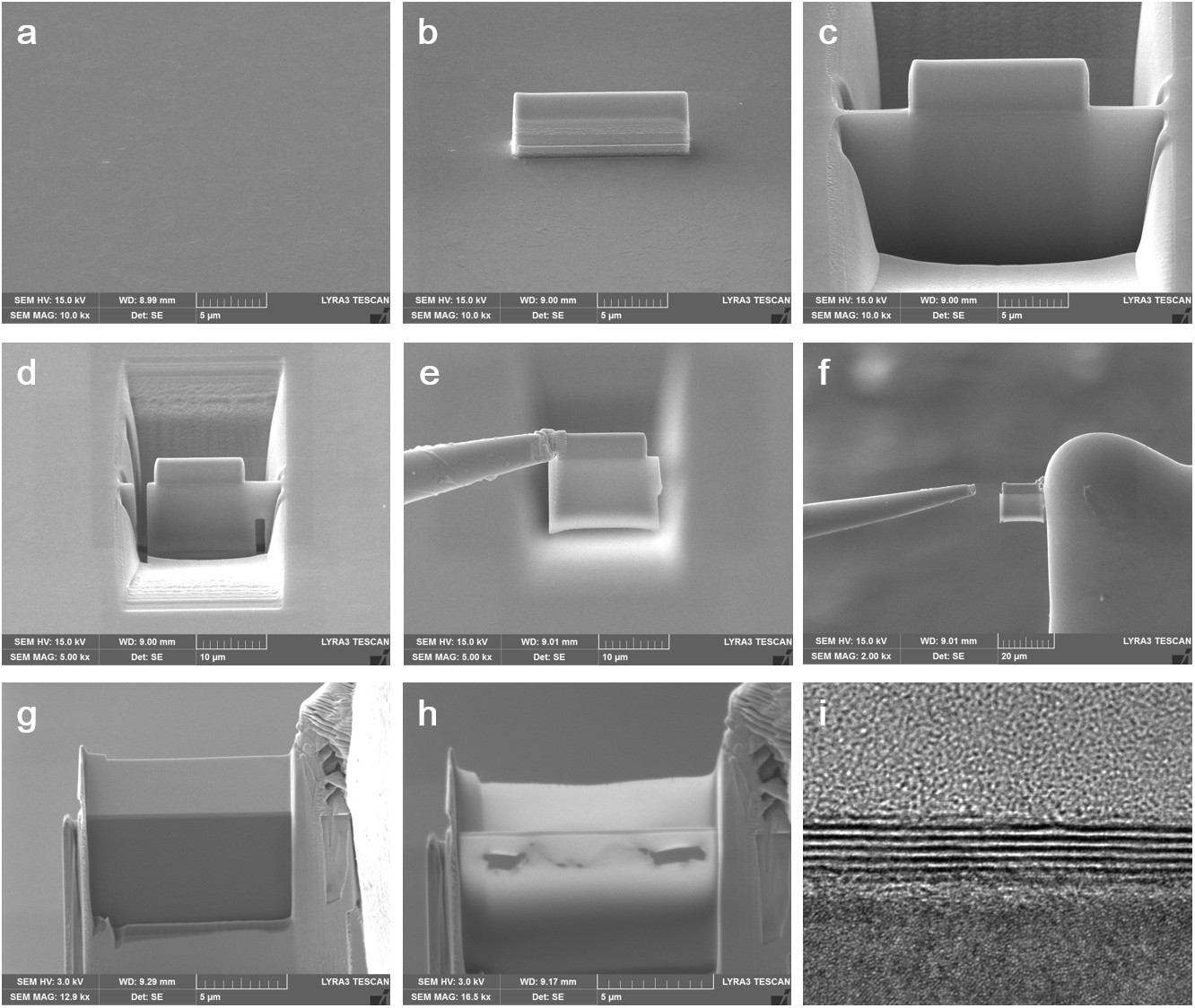


**Figure S8 | Contact angle images** of PbS (a) and MoS2 (b) in their spin-coated state and spin-coated into MoS2/PbS heterostructure (c). MoS2 film after oxygen plasma treatment for 50W with 10 s (d), 15 s (e), and 20 s (f), are presented as well.

**
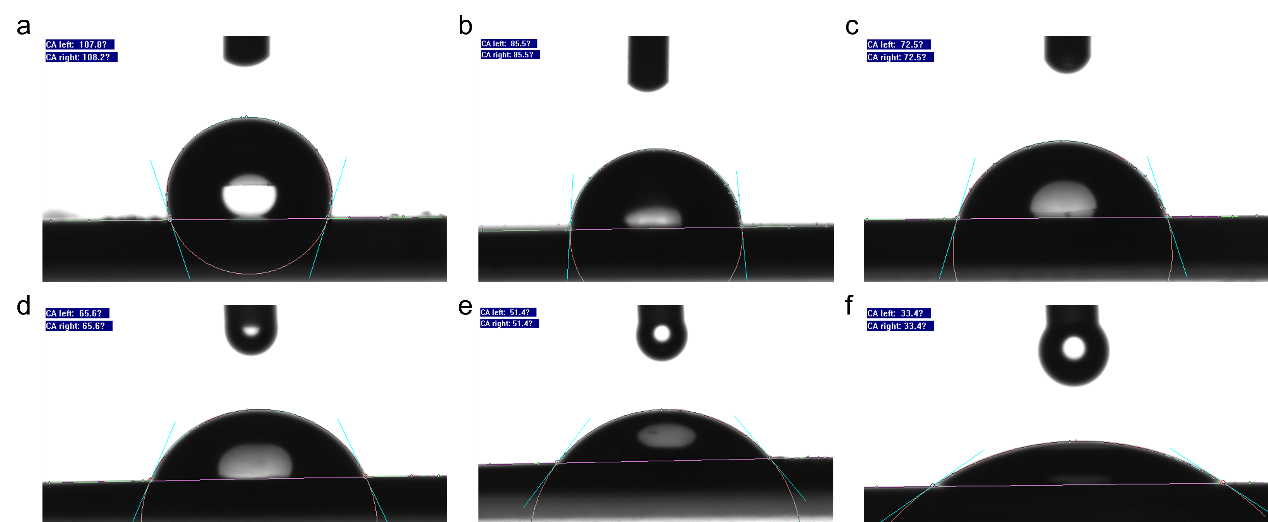
**

**Table S1 | Contact angle revolution of MoS2 under various power and treatment time**

| **Power/W**  **Time/s** | **50** | **75** | **100** |
| --- | --- | --- | --- |
| **5 s** | **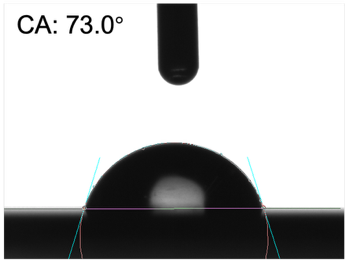** | **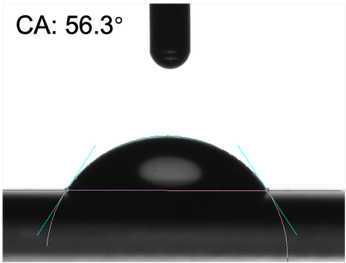** | **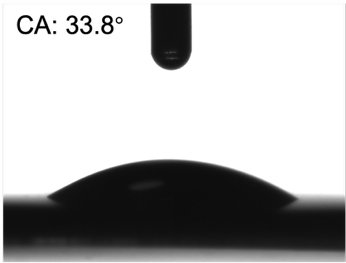** |
| **15 s** | **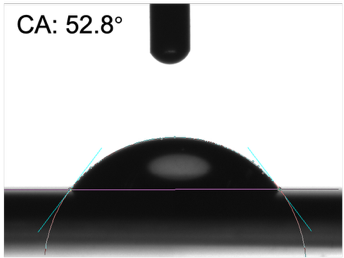** | **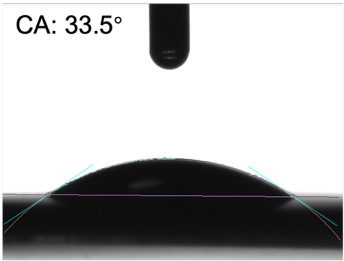** | **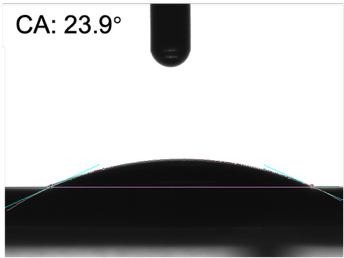** |
| **30 s** | **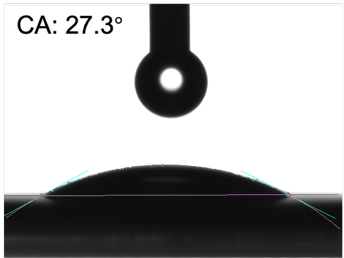** | **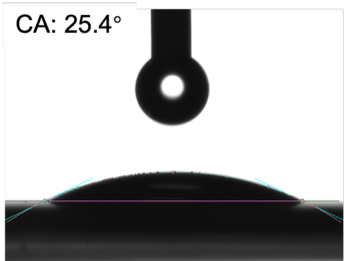** | **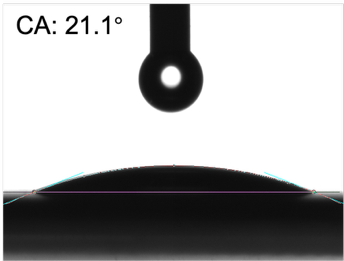** |
| **60 s** | **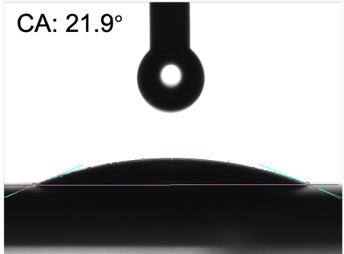** | **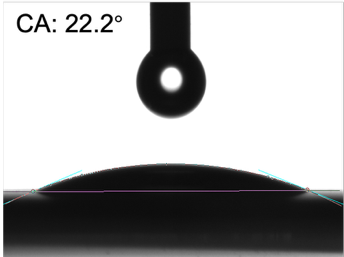** | **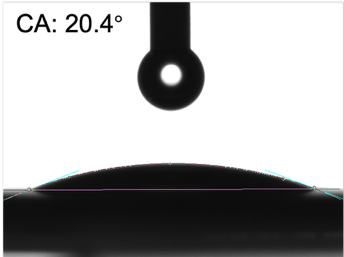** |

**Figure S9 | Contact angle revolution of MoS2 under various power and treatment time** The light-blue area in the figure denotes the range of ideal contact angles, indicating good surface wettability for the subsequent layer.


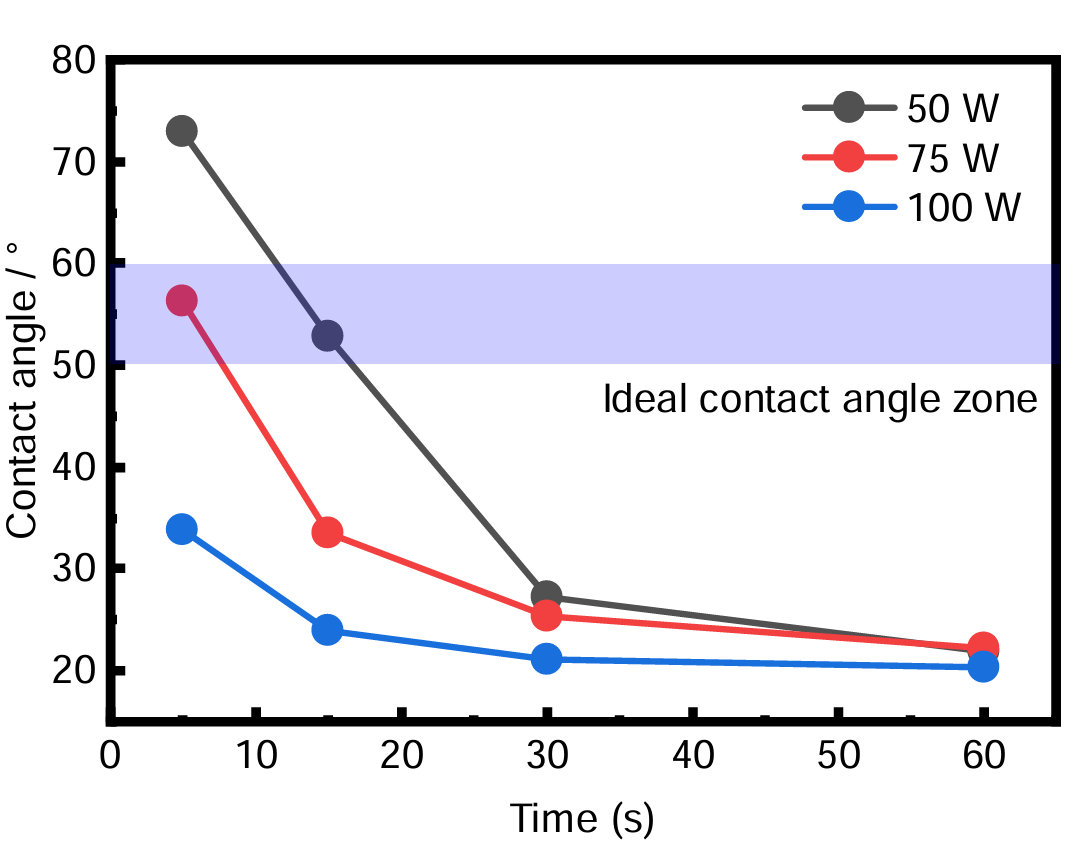


**Figure S10 | Wafer-scale uniformity characterization of wafer 1#** **a,** The five-point measurement positions and their corresponding standard deviations. **b–f,** Transfer characteristics, standard deviations, and yield (100 devices per die) obtained from five individual dies.

**
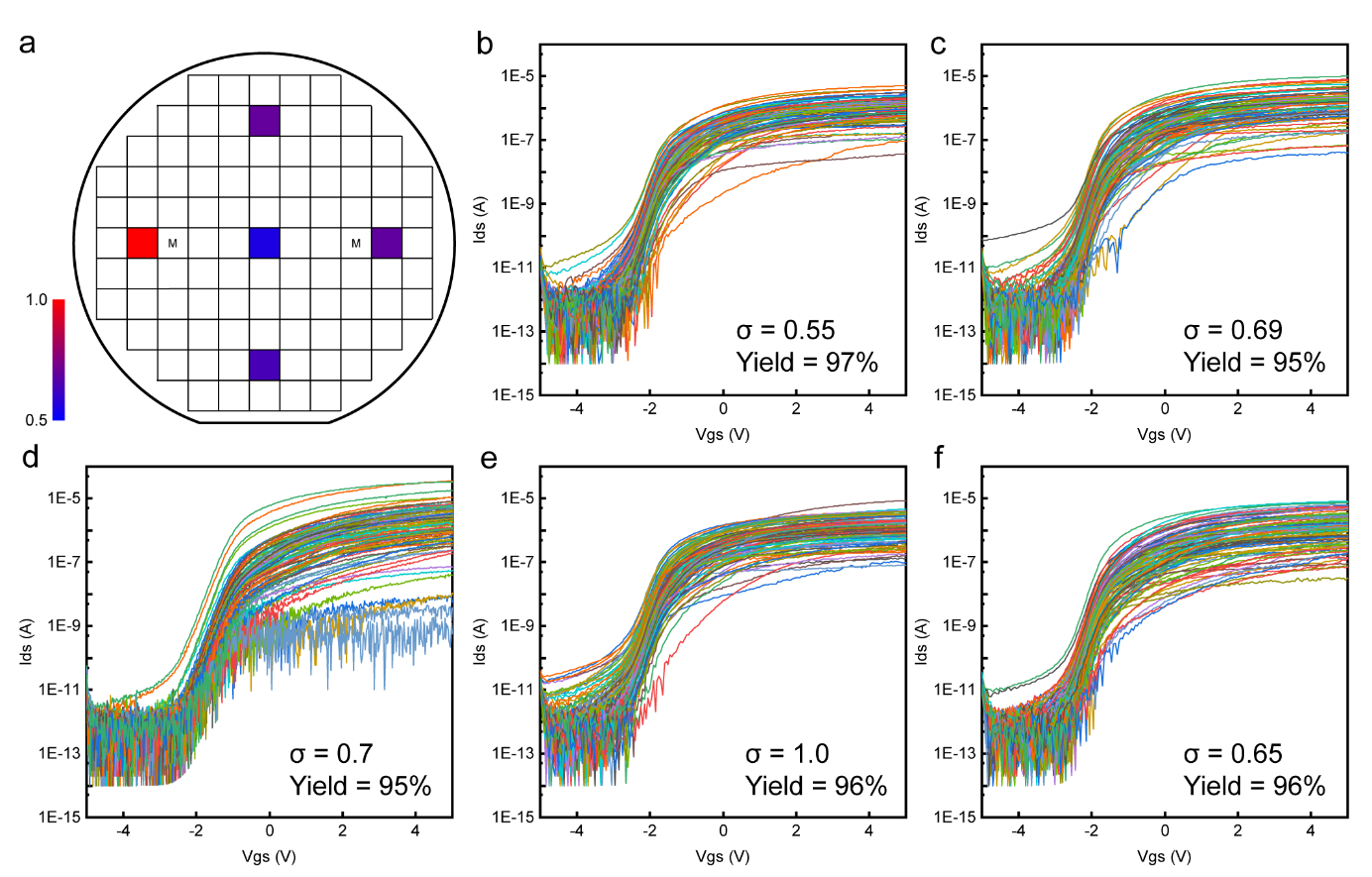
**

**Figure S11 | Statistical distribution of on/off ratios of wafer 1# a,** Combined distribution from all five dies. **b-f,** Individual distributions for each die. A Normal fit was applied to each distribution to extract the mean (representing the central performance value) and standard deviation, σ (indicating the parameter dispersion). The device yield was calculated based on the proportion of devices falling within ±2σ of the mean.

**
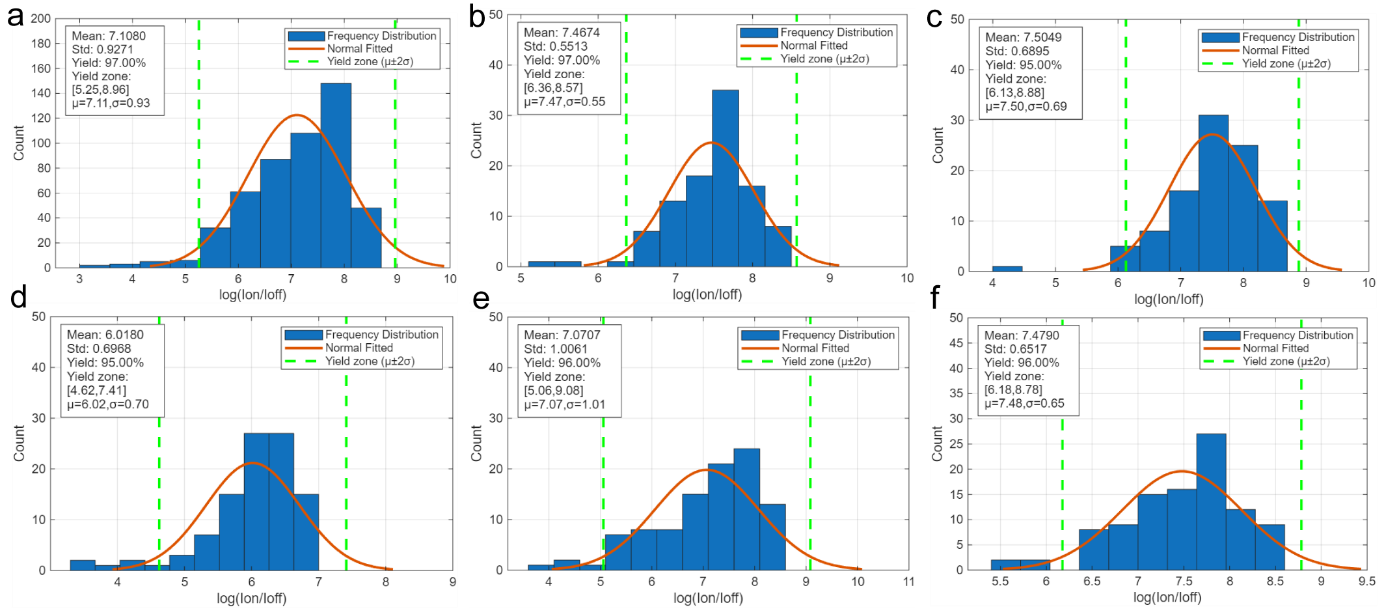
**

**Figure S12 | Wafer-scale uniformity characterization of wafer 2#** **a,** The five-point measurement positions and their corresponding standard deviations. **b–f,** Transfer characteristics, standard deviations, and yield (100 devices per die) obtained from five individual dies.

**
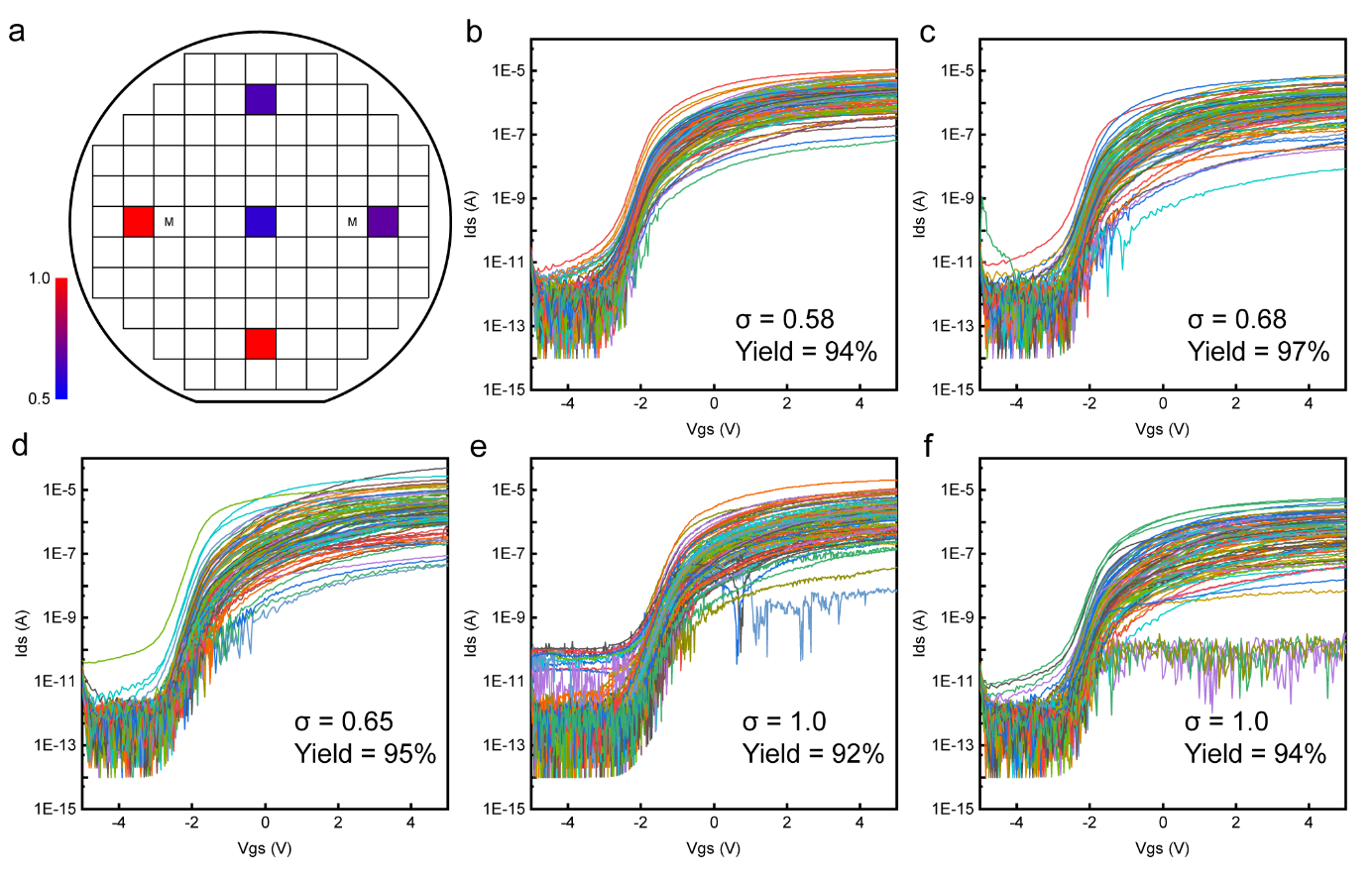
**

**Figure S13 | Statistical distribution of on/off ratios of wafer 2# a,** Combined distribution from all five dies. **b-f,** Individual distributions for each die. A Normal fit was applied to each distribution to extract the mean (representing the central performance value) and standard deviation, σ (indicating the parameter dispersion). The device yield was calculated based on the proportion of devices falling within ±2σ of the mean.

**
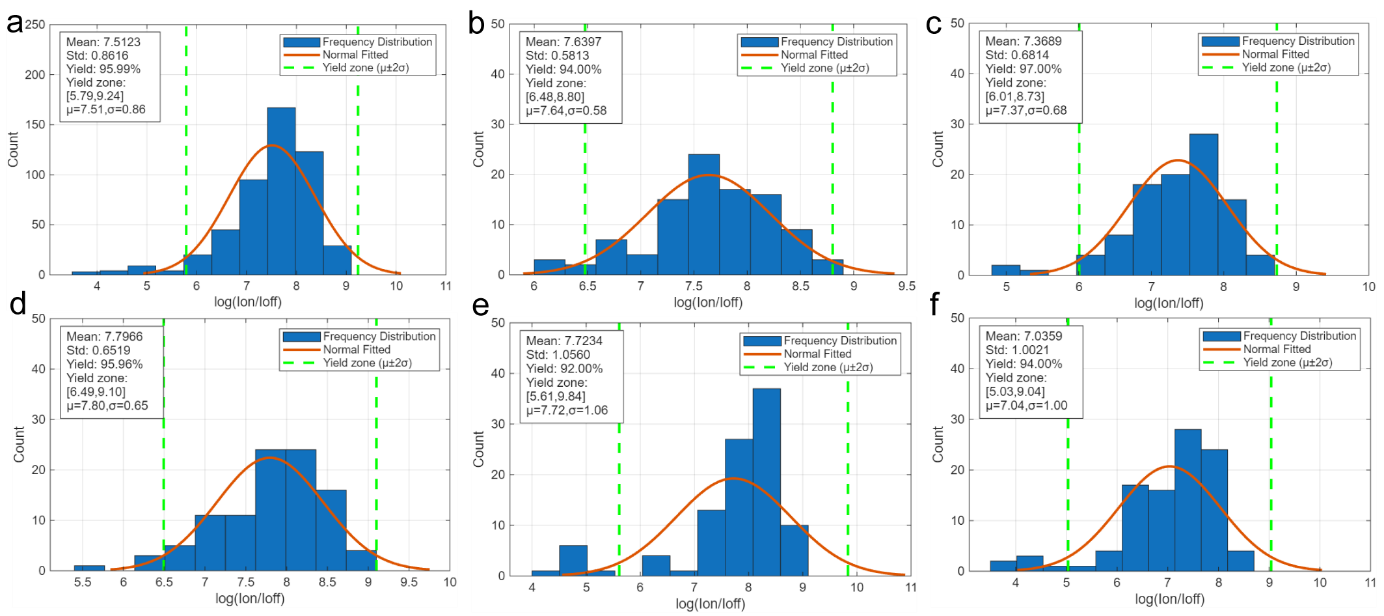
**

**Figure S14 | Statistical distribution of on/off ratios and threshold voltage a-b,** On/off ratios of wafer 1# die 1# and wafer 2# die 2# respectively. **c,d,** Threshold voltage distribution of wafer 1# wafer 2# respectively.

**
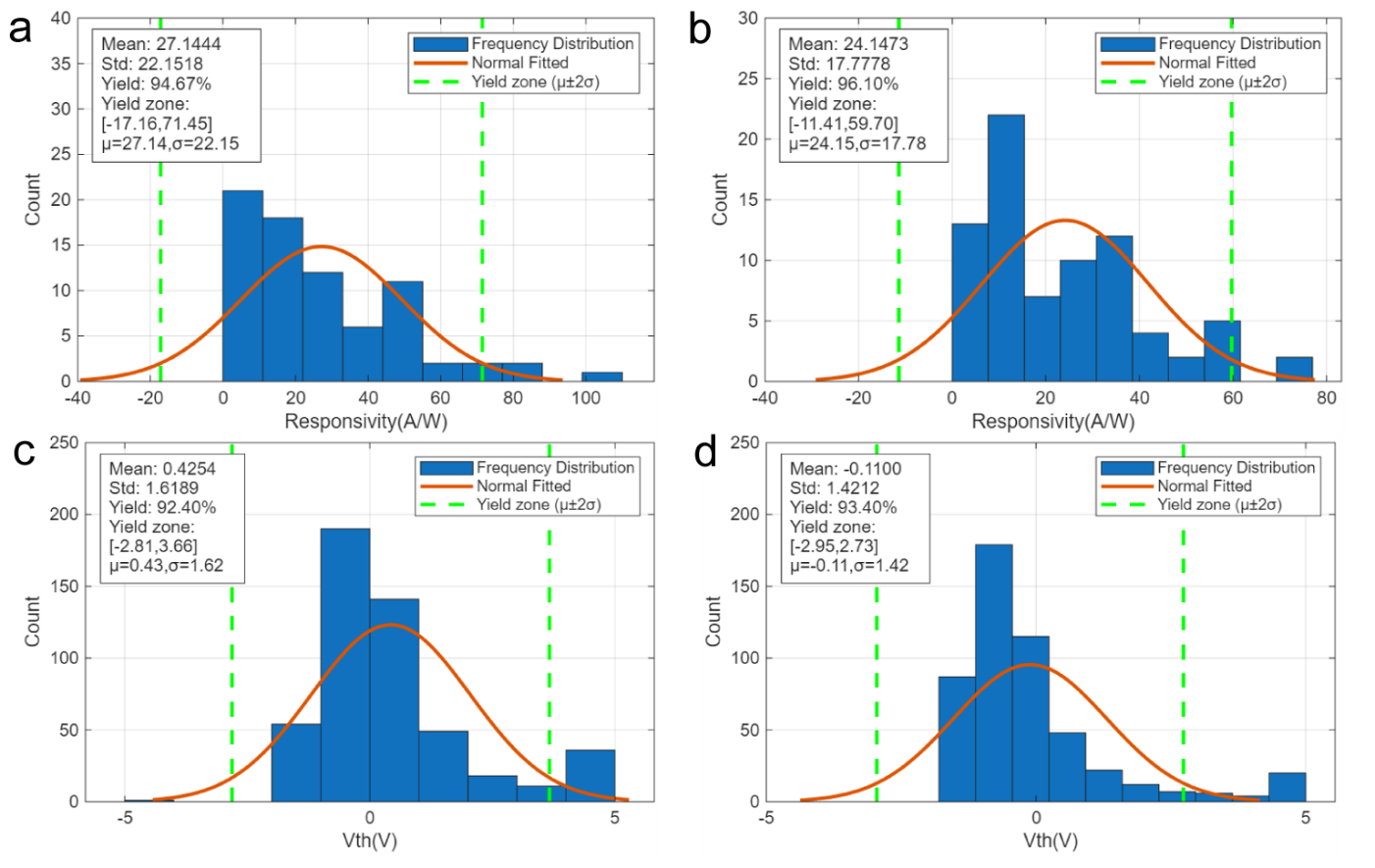
**

**Figure S15 | Device packaged into the CLCC48 configuration** The device was first mounted on a ceramic CLCC48 substrate using epoxy adhesive, and then the electrical connections were established via wire bonding. After encapsulation, the device can be tested without a probe station and can be readily integrated into PCB circuits.

**
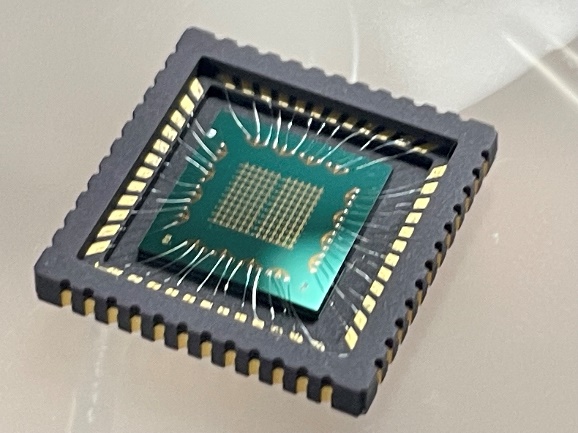

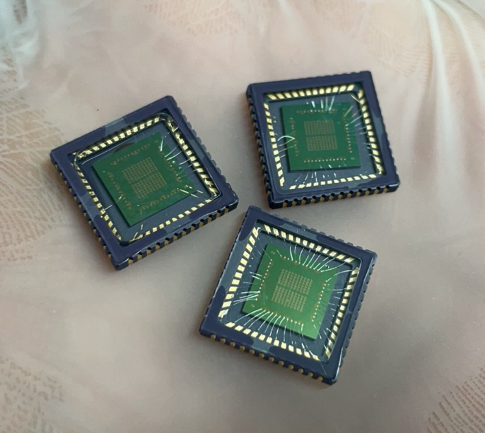
**

**Figure S16 | The transfer curve demonstrates an on/off ratio of 1.8 × 10⁸.**


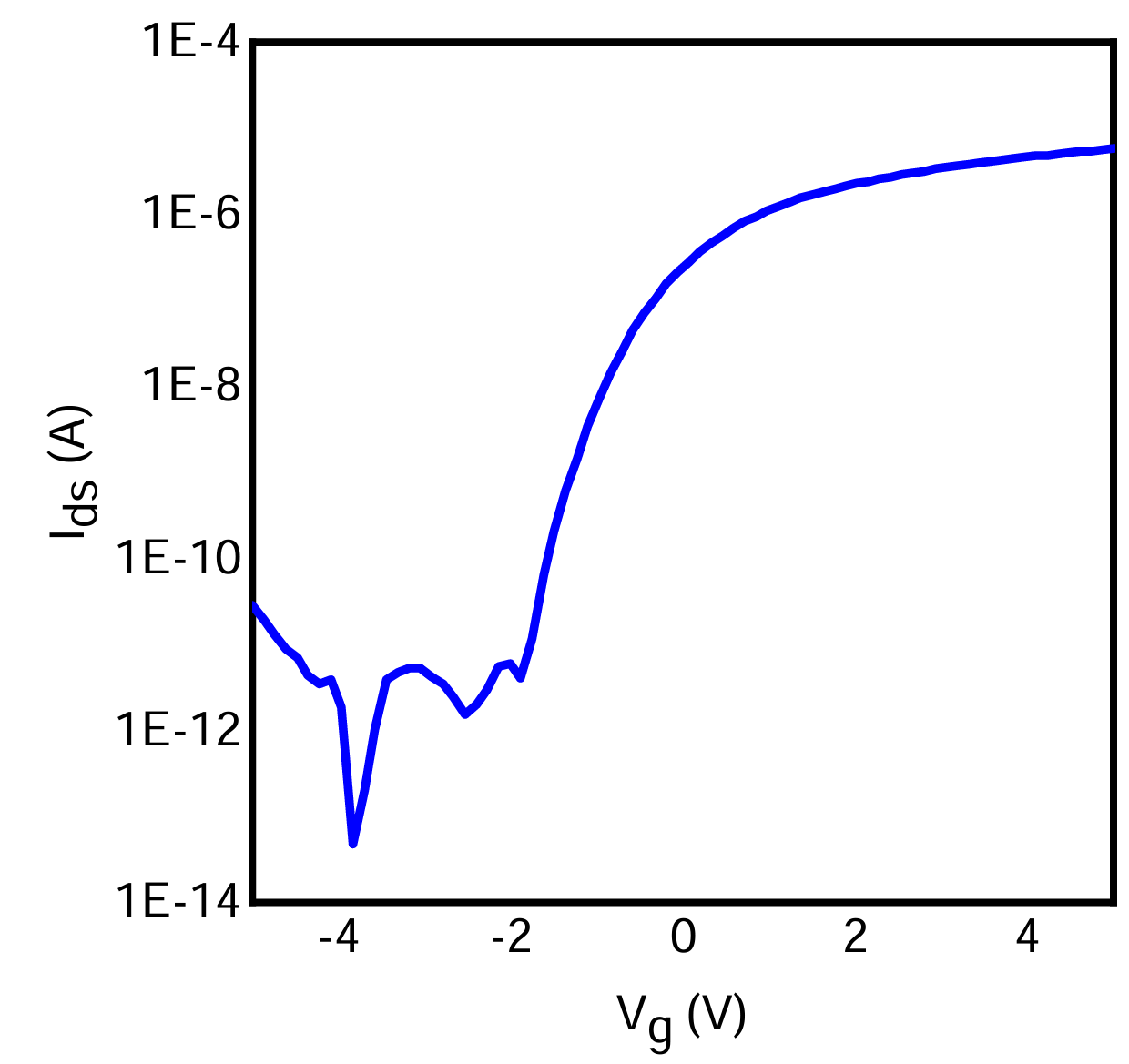


**Figure S17 | Temporal stability of devices with and without AlOx passivation.** The device protected with an AlOx passivation layer (left) maintained good performance after 3 weeks, whereas the device without the AlOx layer (right) exhibited a slight performance degradation.


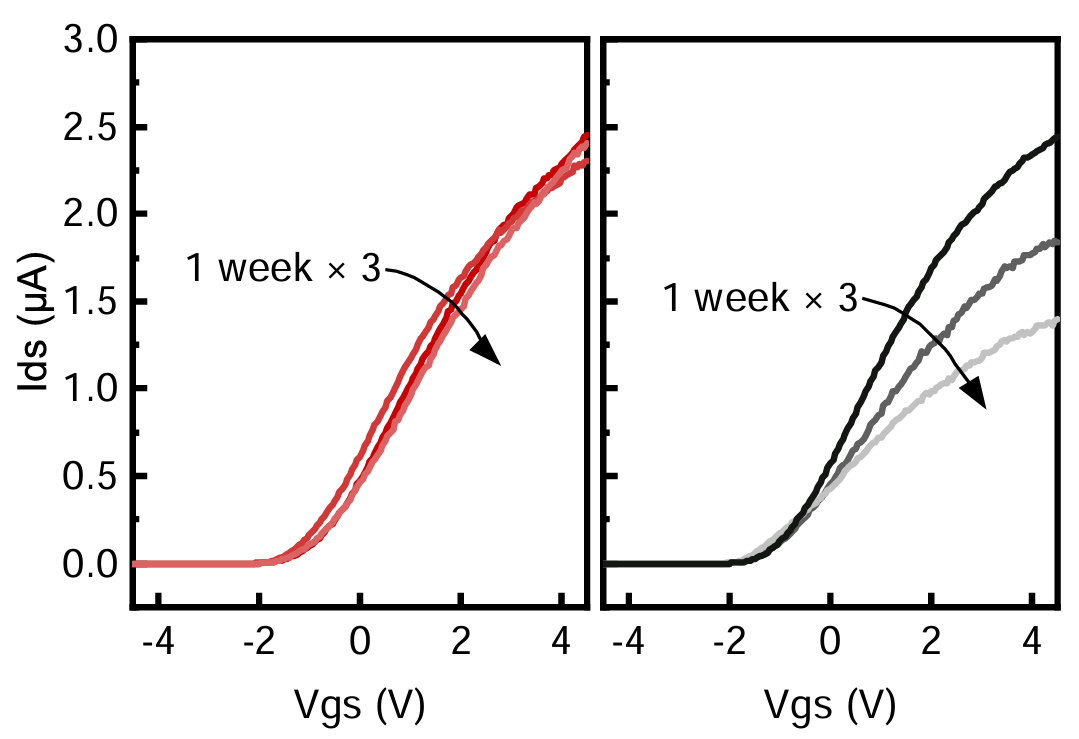


**Figure S18 | Optical scanning imaging test under 850 (top), 980 (bottom) nm**


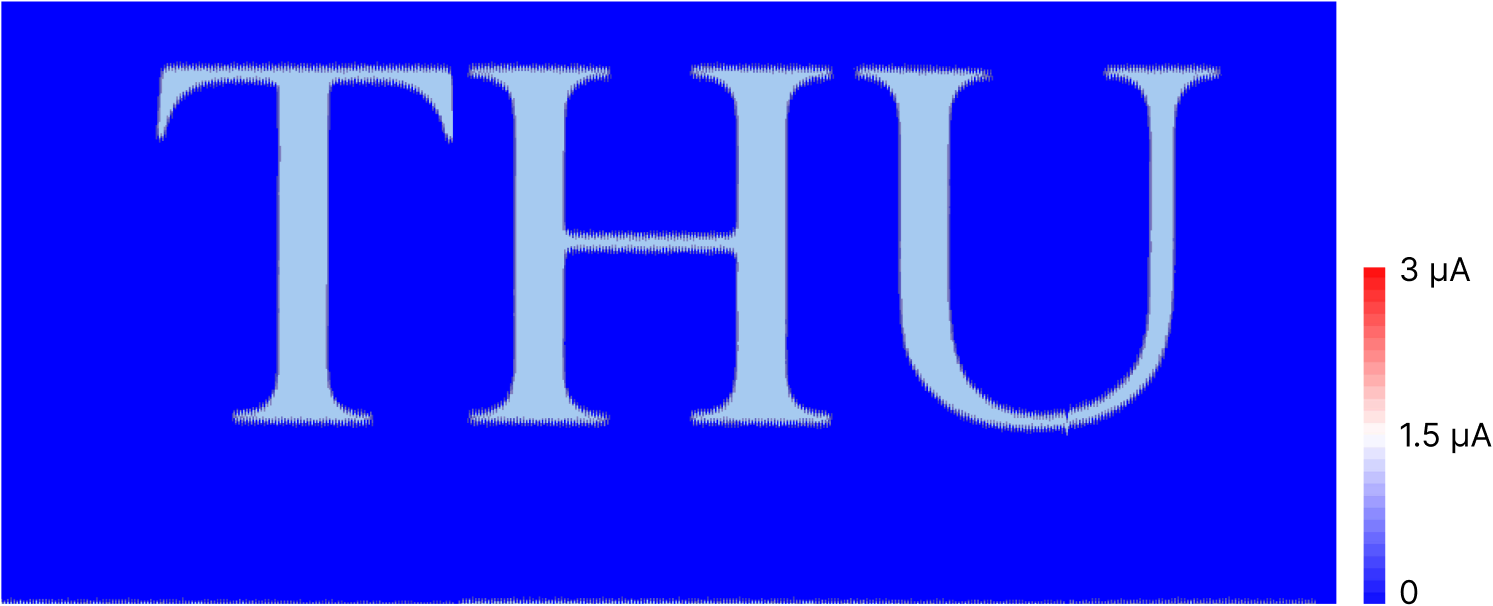


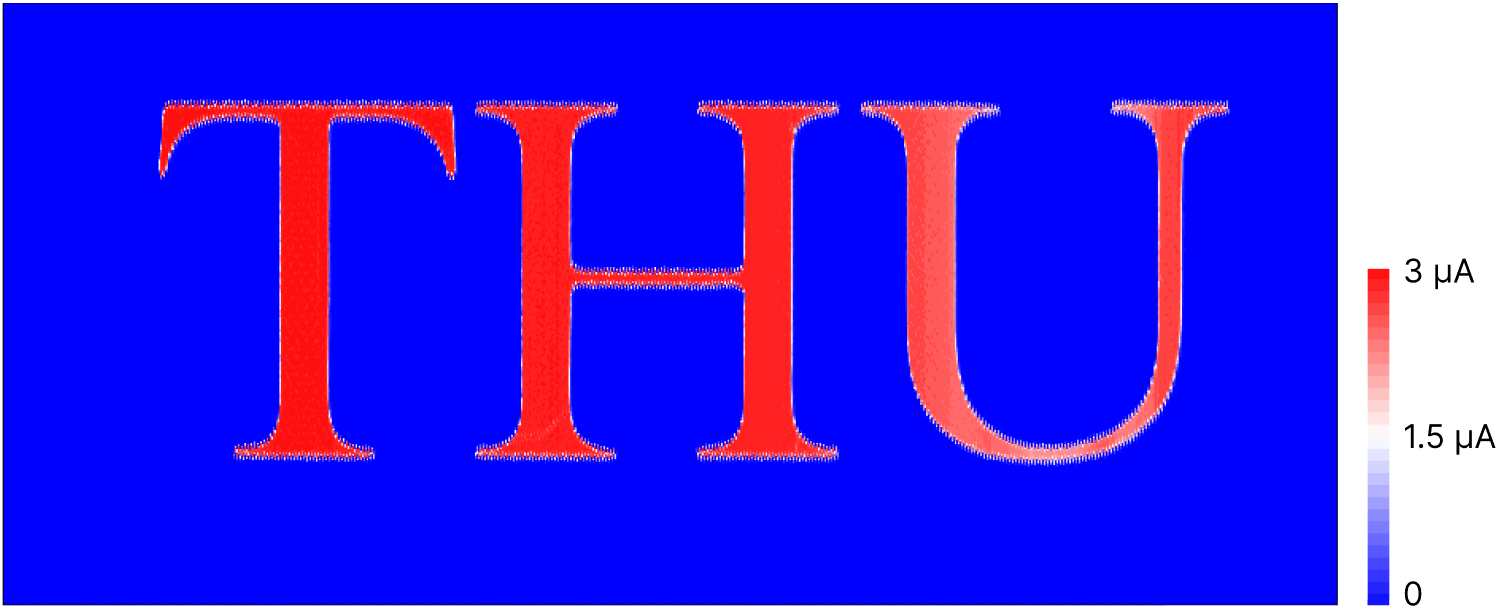


**Table S2 | Comparison of the optoelectronic parameters for relevant photodetectors.**

| Devices | Scale level and Fabrication | Wavelength or Bandgap | On/Off ratio | R [A/W]@ wavelength | D* [jones] | Year^Ref.^ |
| --- | --- | --- | --- | --- | --- | --- |
| MoS2/PbS | 4-inch; Spin coating | 980 nm;  tunable from  1.24 to 0.61 eV | ~10^8^ | 88@  650 nm | 4.77×10^12^ | 2025 This work |
| WS2/ Graphene | ~5×40 μm^2^; Mechanically exfoliated | 1500 nm | 3500 | 0.26  1560 nm | / | 2025^[11]^ |
| PbS QDs | ~ 10×10 mm^2^; Solution Processed | 1240 nm | / | 0.65@  1240 nm | 2.4×10^12^ | 2025^[12]^ |
| BP-MoS2 | ~ 60×60 μm^2^; Mechanically exfoliated | 3600 nm | / | 0.66@  3600 nm | 2.38×10^11^ | 2025^[13]^ |
| Graphene/MoS2 | 3×3 cm^2^;  Transfer after MOCVD | 607 nm | ~10^8^ | 9.37@ 544 nm | / | 2025^[14]^ |
| (PEA)_2_PbBr_4_ | 4-inch; Spray coating | 365 nm | ~10^6^ | 1.5×10^6^@  365 nm | 1.1×10^16^ | 2025^[15]^ |
| BaTiO_3_-Ga_2_O_3_ | ~10×10 μm^2^  Pulsed laser deposition | 265 nm | / | 1.1@  265 nm | 1.2×10^12^ | 2025^[16]^ |

**References**

[1] G. Kresse, D. Joubert, *Phys. Rev. B* **1999**, *59*, 1758.

[2] J. P. Perdew, K. Burke, M. Ernzerhof, *Phys. Rev. Lett.* **1996**, *77*, 3865.

[3] Q. Wang, Y. Wen, K. Cai, R. Cheng, L. Yin, Y. Zhang, J. Li, Z. Wang, F. Wang, F. Wang, T. A. Shifa, C. Jiang, H. Yang, J. He, *Science Advances* **2018**, *4*, eaap7916.

[4] J. Xue, Y. Dai, S. Wang, J. He, T. Xia, J. Hao, Z. Sofer, Z. Lin, *Chem* **2024**, *10*, 1471.

[5] G. Polumati, V. Adepu, C. S. R. Kolli, J. Reji, P. Sahatiya, *Materials Science in Semiconductor Processing* **2023**, *153*, 107161.

[6] C. L. Heideman, S. Tepfer, Q. Lin, R. Rostek, P. Zschack, M. D. Anderson, I. M. Anderson, D. C. Johnson, *J. Am. Chem. Soc.* **2013**, *135*, 11055.

[7] A. Khichar, A. Hazra, *Applied Surface Science* **2025**, *679*, 161252.

[8] L. W. Schwartz, R. V. Roy, *Physics of Fluids* **2004**, *16*, 569.

[9] C. Liu, Y. Jiang, B. Shen, S. Yuan, Z. Cao, Z. Bi, C. Wang, Y. Xiang, T. Wang, H. Wu, Z. Liu, Y. Wang, S. Wang, P. Zhou, *Nature* **2025**, *646*, 1081.

[10] S. Ghosh, Y. Zheng, M. Rafiq, H. Ravichandran, Y. Sun, C. Chen, M. Goswami, N. U. Sakib, M. U. K. Sadaf, A. Pannone, S. Ray, J. M. Redwing, Y. Yang, S. Sahay, S. Das, *Nature* **2025**, *642*, 327.

[11] Z. Zeng, Y. Wang, P. Michel, F. Strauß, X. Wang, K. Braun, M. Scheele, *Nano Lett.* **2025**, *25*, 3497.

[12] S. Chen, H. Zhong, X. Wang, G. Pan, H. Tang, F. Fang, J. Wu, W. Wang, L. Xu, J. Tang, J. Hao, K. Zheng, D. Wu, Z. Tang, L. Zhang, L. Cao, P. Müller-Buschbaum, K. Wang, W. Chen, *ACS Photonics* **2025**, *12*, 879.

[13] J. Wu, J. Zhang, R. Jiang, H. Wu, S. Chen, X. Zhang, W. Wang, Y. Yu, Q. Fu, R. Lin, Y. Cui, T. Zhou, Z. Hu, D. Wan, X. Chen, W. Hu, H. Liu, J. Lu, Z. Ni, *Nat Commun* **2025**, *16*, 564.

[14] B. J. Kim, B. Shao, A. T. Hoang, S. Yun, J. Hong, J. Wang, A. K. Katiyar, S. Ji, D. Xu, Y. Chai, J.-H. Ahn, *Nat Electron* **2025**, *8*, 147.

[15] Y. H. Lee, W.-J. Lee, G. S. Lee, J. Y. Park, B. Yuan, Y. Won, J. Mun, H. Yang, S.-D. Baek, H. Lee, J. H. Oh, T. J. Pennycook, G. Kim, J. Mei, L. Dou, *Advanced Materials* **2025**, *37*, 2417761.

[16] H. Wu, L. Shu, Q. Zhang, S. Sha, Z. Liu, S. Li, S. Yan, W. Tang, Y. Wang, Z. Wu, K. Lin, Q. Li, J. Miao, X. Xing, *Advanced Materials* **2025**, *37*, 2412717.

Movie S1.

The fast response time (ms-level) of the device to light illumination is demonstrated.
